# An effective and novel pore sealing agent to enhance the corrosion resistance performance of Al coating in artificial ocean water

**DOI:** 10.1038/srep41935

**Published:** 2017-02-03

**Authors:** Han-Seung Lee, Jitendra Kumar Singh, Mohamed A. Ismail

**Affiliations:** 1 Department of Architectural Engineering, Hanyang University, 1271 Sa 3-dong, Sangrok-gu, Ansan 426-791, Korea; 2 Department of Chemistry, Indian Institute of Engineering Science and Technology (IIEST), Shibpur, Howrah 711 103, West Bengal, India; 3 Department of Civil and Construction Engineering, Faculty of Engineering and Science, Curtin University Sarawak, CDT 250, 98009 Miri, Sarawak, Malaysia

## Abstract

A new technique was accepted to fill the porosity of Al coating applied by arc thermal spray process to enhance corrosion resistance performance in artificial ocean water. The porosity is the inherent property of arc thermal spray coating process. In this study, applied coating was treated with different concentrations of ammonium phosphate mono basic (NH_4_H_2_PO_4_: AP) solution thereafter dried at room temperature and kept in humidity chamber for 7d to deposit uniform film. The corrosion resistance of Al coating and treated samples have been evaluated using electrochemical impedance spectroscopy (EIS) and potentiodynamic techniques with exposure periods in artificial ocean water. Electrochemical techniques, X-ray diffraction (XRD), Raman spectroscopy, atomic force microscopy (AFM) and field emission-scanning electron microscopy (FE-SEM) indicated that phosphate ion would have been retarding corrosion of Al coating effectively. The formation of AHP (Ammonium Aluminum Hydrogen Phosphate Hydrate: NH_4_)_3_Al_5_H_6_(PO_4_)_8_.18H_2_O) on Al coating surface after treatment with AP is nano sized, crystalline and uniformly deposited but after exposure them in artificial ocean water, they form AHPH (Aluminum hydroxide phosphate hydrate Al_3_(PO_4_)_2_(OH)_3_(H_2_O)_5_) that is very protective, adherent, uniform and plate like morphology of corrosion products. The AHPH is sparingly soluble and adherent to surface and imparted improved corrosion resistance.

Extensive studies are being carried out to protect the steel in different aggressive environments i.e. Cl^−^, CO_3_^2−^ and SO_4_^2−^ etc. The corrosion of steel is a continuous process which cannot be stopped but it can be minimized or slow down/reduced by using different protective schemes. Hence, different types of protective schemes are being adopted to control the corrosion of freshly erected or rusted surface after their proper cleaning. These are included galvanized coating, use of corrosion resistance steel alloys, corrosion inhibitors, cathodic protection, use of organic/metallic and thermal spray coatings. Among these processes, the thermal spray coating is convenient and feasible with minimum drawbacks^1^,^2^. Different thermal spray process had developed i.e. flame, arc thermal, plasma spray, high velocity oxy fuel etc. The arc thermal spray process is convenient, feasible, and economical. The major advantage of arc thermal spray over other process is the wide range of materials that can be used for the coating. Other advantages of thermal spray coating process included that it can be used without decomposing the properties of material and deterring the mechanical and chemical properties of substrate. Such type of coating is wear and corrosion resisting and it can be applied on large structures too.

In electric-arc (wire-arc) spray process metal is being used in wire form. Heating and melting occur when two electrically opposed charged wires comprising the spray material, are fed together in such a manner that a controlled arc occurs at the intersection. The electric-arc process is in most instances less expensive to operate than the other processes. The electric-arc process mostly uses relatively ductile, electrically conductive wires. By using dissimilar wires, it is possible to deposit pseudo alloys.

In arc thermal spray process molten metal on the wire tips is atomized and propelled onto a prepared substrate by a stream of compressed air^3^. After process of coating, a diffused layer formed on substrate. During solidification and diffusion of metals towards substrate, some pores/defects are being formed on coating. The pores/defects are in different sizes, interconnected to each other and it promotes to get moisture, oxygen and other aggressive ions from atmosphere. Due to the high speed of spraying and sudden cooling of melted metal droplets spread at the substrate, therefore, some invisible porosity in the deposited coating and shrinkages are developed^1^,^4^,^5^,^6^,^7^.

It has been reported that the arc thermal spray process coating is having inherent properties such as formation of splash zones and pores/defects on coating surface^8^,^9^. These pores/defects are interconnected to each other. To minimize the porosity of coating and maintain the high resistance to corrosion, different techniques have been recommended. Generally, researchers are recommending abrading of roughened coating, application of epoxide or polymeric coatings to fill the pores of arc thermal sprayed coating^10^.

Many types of sealers have been examined, including polyurethane, phenolic, epoxy, wash primers, inorganics and silicates that are readily available and easily applied on coating surface. Epoxy and phenolic sealants are usually more effective on coatings with less porosity. Such dual (metallic-polymeric) coatings initially exhibit good performance but after their longer duration of exposure in aggressive environment, develop cracks and pores on surface. Unfortunately, the sign of distress on the surface of coating with polymer appeared within 10 years of their construction^11^. The findings of the investigation of distressed structures during their service life were so alarming that many experts are not recommending the polymeric coating applied on metallic surface^12^,^13^. It is due to a vast difference in contraction and expansion coefficients of metallic and polymeric materials. Moisture, aggressive acidic gases and oxygen migrate towards steel surface from coating and cause distress to the structures due to crevice and pitting corrosion. To overcome this problem and block the pores/defects of coating some chemical treatment would be useful to enhance the corrosion resistance properties of coating. *Zhang et al*. have discussed about the pore sealing process for AZ80 Mg alloy to enhance the corrosion resistance performance for biomedical application in saline environment and they used hierarchical coating composed of an inner compact hydroxide layer and top uniform Mg-Al layered double hydroxide micro sheets prepared by a one-step hydrothermal process in water^14^. Robust, self-healing and super hydrophobic fabrics was prepared by *Xue et al*. to enhance the abrasion resistance for clothing but it has limited application and not being possible to use in metal coating^15^. The corrosion resistance properties of synthesized super hydrophobic materials are encouraged by formation of lotus leaves microstructure on surface which repel the water molecules significantly and reduces the affinity with metal surface^16^. However, the paper cited in refs 14, 15, 16 did not describe about the feasibility, long term exposure in aggressive environment for corrosion assessment, environmental issues and cost of process. Therefore, it is very important to take precaution of chemical treatment which would be compatible, feasible and environmental friendly to particular metallic coating.

Literature search revealed that very limited information is available on the use of fillers or other techniques to block the porosity of arc thermal sprayed coating. A US patent published in 2007 about reducing the porosity of thermal sprayed coatings by using different inorganic salts for different metallic coatings i.e. yttrium salt for yttrium coatings, aluminum salt for alumina coatings, zirconium salt for zirconia coatings and such treatment can be employed on other metallic coatings too^17^ but there was no study that had been performed on corrosion characteristics in aggressive or accelerated environments. Another US patent is also explained the pore sealing process of electroplated and chemical plated metallic coating system by using multiple steps with different chemical ingredients^18^. Such process needs more technical schemes, chemicals and scientific knowledge. *Lee et al*. studied the pore blocking effect of Al coating in industrially polluted and 3.5 wt.% NaCl solution with exposure periods^8^,^9^. They found that the corrosion products itself block the pores/defects of Al coating which was applied by arc thermal metal spray process on mild steel substrate after exposure in aggressive environment.

Currently, researchers are using different pseudo alloys and rare earth metal in coating to enhance the corrosion resistance properties of steel in aggressive environments^19^,^20^. Except pseudo alloy, there are different chemical treatments that were also popularized such as chromating, use of arsenic and mercury etc. which have negative effects on human lives but at the same time improved corrosion resistance of substrate was found^21^,^22^,^23^,^24^,^25^,^26^. Some hydrophobic treatment has also been employed on surface but it also has limited stability and defects at molecular level which allow aggressive ions move towards base substrate^27^. *Hughes et al*. suggested about the encapsulation of green inhibitor to heal the defect of coated surface but it needs proper precaution and expertise to develop such process^28^. Such process included polymer which contain encapsulated green inhibitor but it is not possible to use to fill the porosity of coating. However, it is very hard to accept such system and most importantly that this coating could not be used without polymer and it is already discussed on above paragraphs about the drawback of polymer metallic coating.

The phosphate conversion coating is popularized due to its non-conducting, hard in nature and its ability to improve the corrosion resistance properties of materials^29^. Phosphate coating is being used in different sector i.e. automobile and industries due to low cost, rapid coating formation and applied easily on surfaces^30^. After adding of alumina in phosphate chemical conversion coating through ultra-irradiation, it enhances the corrosion resistance properties of steel substrate because alumina alters the preferential growth of direction of crystal and reduces the crystal size^31^. The use of phosphate salts does not have any harmful effects on lives and it is economical. Phosphate treatment for different metallic surfaces such as steel and aluminum, works as conversion coating. The application of phosphate is known for corrosion and wear resistance.

From the above literature search, it is found that no study had been carried out to fill the pores of metallic coating using arc thermal spray process by chemical treatment. Hence, it is established that the best suitable treatment of coating with phosphate containing salts to be required which has high affinity to metallic surface and may fill the porosity of coating. In this study, treatment of phosphate on arc thermal sprayed Al coating by dissolving phosphate salt in double distilled water using brush was performed. Such process is very easy, convenient and eco-friendly to treat the defective coating surface deposited by arc thermal spraying process. The phosphate reacts with Al metal and formed a protective layer of metal-phosphate and fill the pores of coating which enhances the corrosion resistance properties in aggressive environments.

From the above mentioned discussion, it is clear that to fill the pores of Al coating applied by arc thermal spray process on steel surface needs proper attention. In present study, different concentrations of phosphate containing salt has dissolved in water and corrosion resistance performance of treated coatings by different electrochemical techniques in ASTM D1141 solution^32^ was evaluated. The electrochemical techniques suggest about the kinetics and mechanism of corrosion process. Such treatment process is very easy to apply on metallic surface, versatile, environmental assisted, favorable and has commercial application without using any resin or hydrocarbon. The solid compound of AHP uniformly fill the interconnected pores of Al coating applied by arc thermal spray process. This treatment process included dissolution of AP salt in water, solution applied by using brush, treated samples kept in humidity chamber which identified as environmental condition and generate natural pore sealing solid compound. Finally, such treated surface reacts with artificial ocean water and formed very protective and sparingly soluble solid compound which significantly enhances the corrosion resistance of treated coating even in aggressive environment for their longer duration of exposure.

## Methods and Materials

### Process of coating

Aluminum metal was used as coating materials due to its high strength, good mechanical properties and low cost on steel surface by arc thermal spray process. The coating was applied on 80 mm × 60 mm × 1 mm dimensions of sand blasted mild steel substrate containing C = 0.20, Mn = 0.95, Si = 0.26, P = 0.02, S = 0.01, Cu = 0.02, Cr = 0.04, Ni = 0.03, Fe = balance in wt.%. The commercially pure 99.95 wt% of 1.6 mm Al wire had used for this coating. Prior to coating, steel substrate was properly pickled with 10 v/v% HCl, washed with distilled water thoroughly and dried at 25 °C (±1 °C). The coating thickness was measured with non-destructive technique using Elcometer456 at different locations and it was approximately 100 μm (±5 μm).

The process of coating was mentioned in our earlier publications^8^,^9^. After coating, the adhesion test was measured according to ASTM D4541 by selecting 4 cm × 4 cm dimension of coating^33^. The selected surface area for adhesion of coating was much higher than standard. Hence, it would affect the adhesion of coating. The selection of higher coating surface area for adhesion results in lesser adhesion value. The average bond strength for 4 samples of Al coating are 4.86 MPa and it is due to selection of 16 cm^2^ of coating area which is greater than recommended surface area.

After Al coating on mild steel surface by arc thermal spray process, it was treated with different concentrations i.e. 0.1 M, 0.5 M and 1.0 M of ammonium phosphate mono basic/Ammonium dihydrogen phosphate (NH_4_H_2_PO_4_: abbreviated as AP) solution. It is less viscous, water soluble and easily applied by using brush for three times to ascertain uniform treatment. The solutions were designated as AP1, AP2 and AP3 for 0.1 M, 0.5 M and 1.0 M AP, respectively. The solutions pH was measured at 25 °C (±1 °C) and they were 4.50, 4.29 and 4.17 for AP1, AP2 and AP3, respectively. As the content of AP is increased, the pH is decreased. This may be due to acidic nature of AP. The AP was analytical grade and dissolved in double distilled water. The AP dissolved in water and formed phosphoric acid (H_3_PO_4_) and ammonium hydroxide (NH_4_OH) as reaction products. The phosphoric acid is strong acid while ammonium hydroxide is weak base which influences reduction in pH of the solution. Solution treatment process was applied on Al coating surface after every 8 h of interval and dried at 25 °C (±1 °C) and such process was continued up to 24 h. After proper dryness of coating, it was exposed in humidity chamber at 50 °C and 95% RH (relative humidity) up to 7d to ascertain uniform deposition and reaction of AP with Al coating. Thereafter, it was retrieved from humidity chamber and kept at room temperature (25 °C ± 1 °C) up to 7d.

### Electrochemical studies

The electrochemical studies for all coatings were performed in ASTM D1141 solution^32^. This solution simulates the artificial ocean water. It is believed that ocean water is the most aggressive environment to assess the corrosion characteristics of any coating. For preparation of solution, analytical grade of chemicals was used and dissolved in double distilled water at 25 °C using automatic magnetic stirrer up to 10 min thereafter it was filtered. The pH of solution was maintained 8.20 by adding 0.1 M NaOH as stated in standard.

Prior to start the experiments, the coatings were exposed in ASTM D1141 solution for 30 min and stabilized the potential with potentiostat. These studies were performed by three electrode systems where coated sample work as working electrode (WE), platinum wire as counter electrode (CE) and silver-silver chloride (Ag/AgCl) as reference electrode (RE). The sample holder area of working electrode was 0.78 cm^2^ and it was fixed for all samples^8^.

The electrochemical impedance spectroscopy (EIS) studies were carried out by changing the frequency of 10 mV sinusoidal voltage from 100 kHz to 0.01 Hz. DC polarization studies were performed at 1 mV/s scan rate from −0.4 V to +0.8 V Vs open circuit potential^19^,^34^. The potentiostat was VersaSTAT (Princeton Applied Research, Oak Ridge, TN, USA) and data analysis were carried out by Metrohm Autolab Nova 1.10 software by fitting the experimental data in constant phase element (CPE) model. All electrochemical studies were carried out at 25 °C (±1 °C).

After potentiodynamic studies of coatings, the solution analysis was carried out to determine the concentration of leached Al from coating surface. For determination of Al concentration in solution ICP-MS (inductive coupled plasma-mass spectroscopy, SPECTRO-ARCOS FHE16) was used through 165 nm to 780 nm wave length.

### Characterization of coatings and corrosion products

The morphology of coatings and corrosion products was determined by Scanning Electron Microscopy (SEM, Philips XL 30) operated at 15 kV, equipped with an Energy Dispersive X-ray Spectroscopy (EDS) for elemental analysis. Prior to take the SEM images of coatings and corrosion products, they were coated with platinum to increase the conductivity and avoid charging effect.

The atomic force microscopy (AFM) of coatings and corrosion products were carried out by using Park (XE-100) instrument by keeping the samples 12 μm away from the working distance at Z-scanner. The scan range was 20 nm × 20 nm at XY scanner via contact angle mode. The scanner was coupled with X, Y and Z axis. The analysis of AFM results was performed by XE 100 image processing software.

The X-ray diffraction (XRD, Philips X’Pert-MPD) studies of coatings and corrosion products were performed by using Cu Kα radiation (λ = 1.54059 A°) generated at 40 kV and 100 mA.

The Raman spectroscopy (Renishaw RM 1000) of coatings were carried out by using Al-Ga-As diode laser beam of 758 nm wavelength. The power of the laser kept at 10 mW to avoid the transformation of formed phases on coating and corrosion products due to heating effect. The collection time was 10 s and the ranges of Raman shift in between 200 cm^−1^ to 3200 cm^−1^. The locations of the specimens to be studied were focused through an Olympus microscope at the magnification of 20. The sample holder had motorized platform with Jokey to have a fine focusing and mapping at a desired location. Prior to analysis of the samples, the instrument was calibrated by using pure Silicon at the peaks of 520 cm^−1^.

## Results

### Characterization of treated and as coated Al coatings

**Figure 1:**
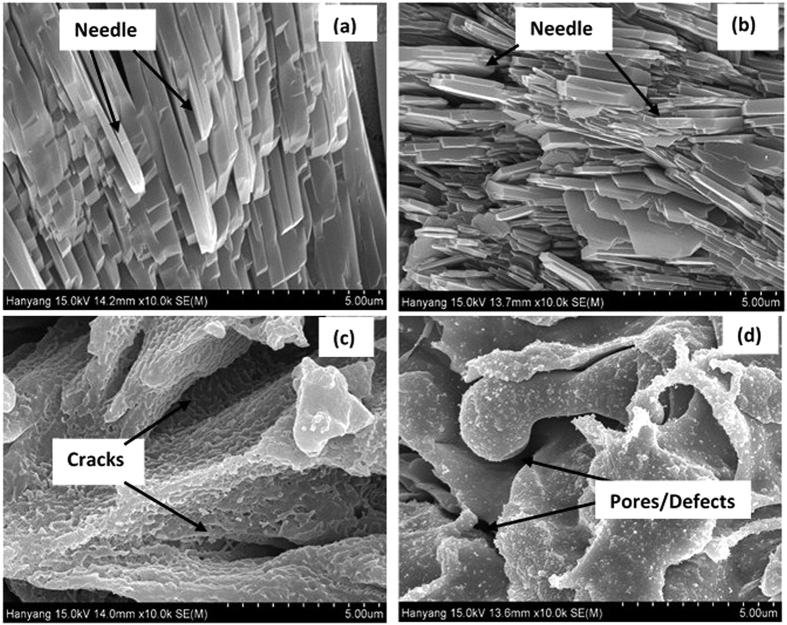
SEM images of Al coating applied by arc thermal spray process **(a)** AP1, **(b)** AP2, **(c)** AP3 and **(d)** AC.

The characterization of AP treated and as coated (AC) Al coating were carried out by different analytical techniques on their top surface. To investigate the microstructure, morphology of AC and added influence of AP solution on treated Al coating of AP1, AP2 and AP3 was carried out by FE-SEM and their respective images are shown in Fig. 1. From this Fig., it can be seen that after treatment with AP; the morphology of Al coating has changed and formed uniform, elongated, filamentous, crystalline and regular needle like structure on AP1 coating without any defect or crack on surface (Fig. 1a). Meanwhile at time of treatment with AP on Al coating surface is differed from each other. The AP1 treated coating has covered all surface with dense, without any visible micro-cracks and pores on microstructure which prevent the penetration of aggressive ions and solution towards coating surface. The AP2 coating (Fig. 1b) exhibited smaller needle and plate like morphology with irregularity in orientation and formed hollow microstructure. The all grown elongated crystals particle of AP1 and AP2 are oriented parallel to each other and formed compact layer over the coating surface. As the concentration of AP solution is increased (AP3) on Al coating it developed cracks (Fig. 1c) which may allow to penetrate the aggressive ions from solution or atmosphere and later it cause deterioration or corrosion of coating. The cracks developed on AP3 treated surface may be due to more content of AP in solution and whatever film had formed is brittle and further it would develop cracking. The chunk type deposition is found on AP3 coating owing to become hard and brittle which makes the coating more susceptible to corrosion^35^,^36^. AC samples are also shown pores/defects on coating surface with plate morphology (Fig. 1d) which is typical properties of arc thermal spray coating process^8^,^9^,^37^. The presence of pores/defects on coating surface allow to penetrate the aggressive species and enhances corrosion of coating. Through pores/defects aggressive species penetrate and substrate become cathode and Al coating work as anode owing to formation of galvanic cell which actively initiate the corrosion process.

The presence of cracks in AP3 and pores/defects on AC surfaces are differed from AP1 and AP2 coating. On the other hand, the affinity of negative phosphate ion of AP solution with positively charged Al is highly appreciated and formed crystalline structures with different morphology due to absorption phenomena^38^,^39^. The content of AP solution plays a major role in morphology of coating. Therefore, the film/layer is uniformly distributed over the coating surface for AP1 and AP2 coating while AP3 shows crack due to higher content of AP solution.

The EDS analysis of coatings were performed along with SEM on captured images. The EDS analysis are shown in Table 1. From Table 1 it is clear that as concentrations of AP solution are increased the content of N, O and P increased. The higher content of N (8.35 wt.%) and P (28.60 wt.%) on AP3 coating surface facilitates the formation of cracking. On the other hand, it might be possible that presence of these elements on coating surface is responsible for reduction in pH of AP solution and development of cracking on AP3 coating (Fig. 1c). On the other hand, there is no other element present except Al and O on AC Al coating.

**Figure 2:**
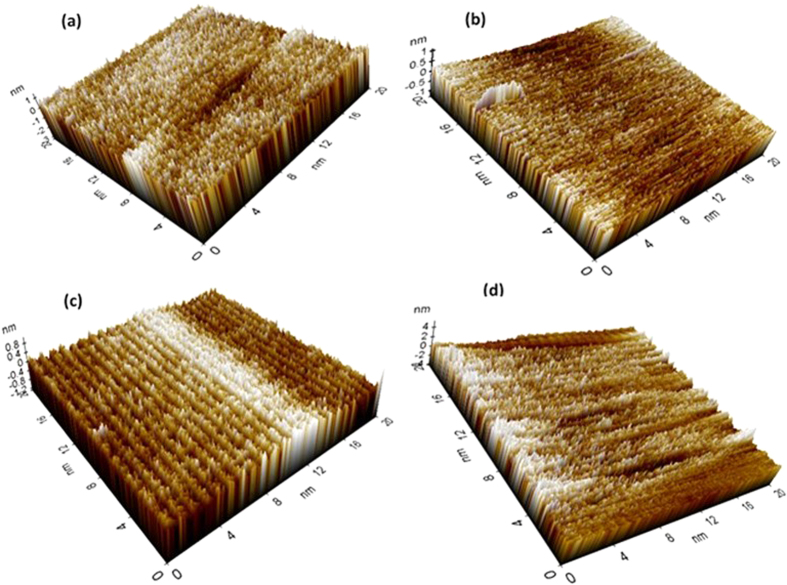
AFM (topography image: 3D) of Al coating applied by arc thermal spray process **(a)** AP1, **(b)** AP2, **(c)** AP3 and **(d)** AC.

The surface topography of treated and AC coating was studied through AFM at 20 nm × 20 nm scan area and shown in Fig. 2. The average roughness of treated and AC Al coatings is measured by using XEI-100 processing software and these are 0.12, 0.46, 0.67 and 0.64 nm for AP1, AP2, AP3 and AC coating, respectively. The treated surface of Al coating is very thin^40^,^41^ except AP3 and AC that exhibited higher roughness due to formation of pores/defects on coating (Fig. 1). It is evident from AFM results that as the concentration of AP solution is increased the roughness of treated surface increased. This result attributed that lower concentration of AP solution exhibited more uniform and homogenous protective film. The AP1 treated coating surface is consisted with less and small number of spikes than other surfaces. The low roughness of AP1 treated sample is considered to be attributed due to the uniform deposition on Al coating surface. As the concentration of AP solution is increased the number and height of spikes increased for AP2 and AP3 coating surface. On AP2 and AP3 surfaces different size of spikes and grooves are randomly distributed. The AP3 treated sample exhibited more number of spikes and ridges on top surface. On the surface of AC Al coating, different types of valley and spikes are present which facilitate the ingress of aggressive species from atmosphere and water molecules^42^. The deposition of salt as can be seen in AFM topography is more on AP3 surface followed by AP2 and AP1 while AC Al coating exhibited different topography of surface than treated coatings. Therefore, this result suggests that AP3 and AC is exhibited high roughness which enables the coating for ingress of aggressive ions^43^. The high roughness of AP3 coating might be due to high content of AP solution which was deposited on surface of Al coating and thus lead to cracking^44^. The AP1 and AP2 are showing less roughness and this means that these coatings would provide better protection against corrosion in aggressive environment^42^.

**Figure 3:**
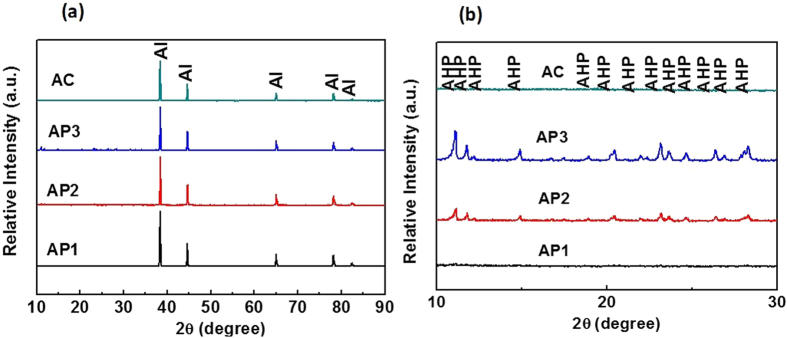
XRD **(a)** full range 2θ = 10° to 90° **(b)** 2θ = 10° to 30° of treated and AC Al coating applied by arc thermal spray process.

The XRD of treated and AC Al coatings are shown in Fig. 3. For all coatings, it is found that Al peaks are prominent and AP treated samples showed very less intensity of other peaks which had formed during treatment. The intensity of other phase is also depending on the concentration of treated chemicals. The presence of Ammonium Aluminum Hydrogen Phosphate Hydrate ((NH_4_)_3_Al_5_H_6_(PO_4_)_8_.18H_2_O: AHP) on treated Al coatings reveled the role of phosphate ions with Al. The formation of AHP can be explained by following equation:


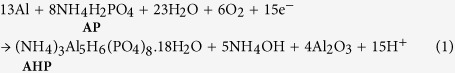


The Al reacted with AP solution and formed AHP on coating consequently release of H^ + ^ion in equation 1 and deposits on surface. However, as can be seen from equation 1 that if the concentration of AP solution is more, the deposition of H^ + ^ion on treated Al coating is more i.e. AP3 treated sample (Fig. 3). The AHP is hydrated Aluminum phosphate and it is soluble in water. Since, the AHP dissolves in the water or moisture and reduces the pH resultant leads to destabilized the coating^45^.

The higher intensity of Al phase suppressed the other peaks in low intensity of AHP in full range of XRD plots (2θ = 10° to 90°). Hence, it is replotted from 2θ = 10° to 30° in Fig. 3b. From Fig. 3b, it is clearly seen that as the concentration of AP solution is increased, the peak intensity of AHP increased which shows the role of AP solution. The AP1 coating shows very less intensity of AHP than AP2 and AP3. It is also clear that the presence of AHP on AP1 surface is very less while on AC Al coating there is no other peak found except Al.

From Table 1 it can be seen that the O content is very less for AC Al coating. This content of O reacts with Al and form different phase of Al oxides/hydroxides. The thickness and content of other formed phase of Al is very less which would not be detected by XRD and it is beyond the limit of instrument. The XRD can detect up to 0.2 μm thickness of film^46^. The penetration of XRD beam is more (it cannot detect low thickness and content of Al phases) due to use of 40 kV and 100 mA power which penetrate towards substrate therefore, the presence of intense peak of Al (Fig. 3) is detected for all samples. Due to penetration of X-ray beam beyond the thin layer^47^ of treated surface only Al is detected but the AHP is found to be more if the content of AP solution is high. However, the thickness of treated samples is in nm which is very less and cannot be detected by XRD. The AHP in AP1 coating is very less therefore, it was not properly detected by XRD^48^. Although we have calculated the volume fraction of AHP and Al on treated coating surface by using integrated surface area calculation^49^,^50^. Based on integrated surface areas, the volume fraction (V_f_) of AHP and Al were calculated^51^,^52^ using following equations:


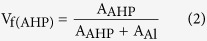



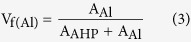


where V_f(AHP)_ and V_f(Al)_ are volume fractions and A_AHP_ and A_Al_ are the total integrated areas, corresponding to AHP and Al phases, respectively. Based on calculations, 4.72, 7.39, 14.42, 0.00% of AHP and 95.28, 92.61, 85.58 and 100.0% of Al for AP1, AP2, AP3 and AC Al coating, respectively were found. As the concentration of AP solution increased, the V_f_ of AHP increases. Since, the content of N, O and P is increased on treated samples, the peak intensity for AHP increased (Fig. 3b). From Fig. 3, it is clear that AP1 treated coating is containing very less amount of AHP (4.72%) while AP2 and AP3 contain higher than it.

The Raman spectroscopy of treated and AC coating are shown in Fig. S1 (Fig. supporting1). Although it is not confirmed by Raman spectroscopy that whether AHP is formed or not but XRD and equation 1 illustrate the presence of it.

From literature search, it is found that no one has reported about the peaks of AHP by Raman spectroscopy. The P-O-P group usually appeared in regions at 1000–1200 and 450–600 cm^−1^ which belongs asymmetric stretching and bending vibrational frequencies, respectively and in this study 1110 and 450–550 cm^−1^ appeared. The presence of P-O-P group around such vibrational frequency attributed the formation of phosphate containing compounds. The Al-O-Al groups for asymmetric symmetry, symmetric stretching and asymmetric bending vibration around 850–950, 650–850 and 200–300 cm^−1^, respectively^53^,^54^,^55^ corroborate our findings around 960, 850 and 225–310 cm^−1^ for all treated Al coatings. The other symmetric stretching ν_as_Al-O-P around 720 cm^−1^ is found which indicates the presence of Al-O-P group in AHP. The Raman shift at 1260 cm^−1^ attributed due to formation of η-Al_2_O_3_ which might be not detected by XRD due to very less content and unstable^56^. In AHP, the NH_4_^+^ ion does not rotate and induce to generate induced dipole moment after apply of vibrational frequency. Therefore, it does not show any peaks in Raman spectroscopy study.

### Corrosion resistance properties evaluated by different electrochemical techniques

#### Potential time studies

**Figure 4:**
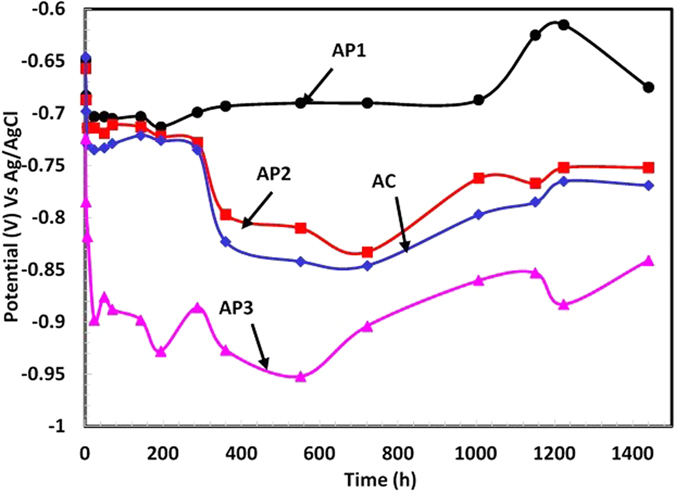
Potential time plots of treated and AC Al coating applied by arc thermal spray process in artificial ocean water with exposure periods.

Corrosion resistance properties of AP treated and AC Al coating were evaluated in artificial ocean water with their exposure periods at their open circuit potential (OCP). The corrosion potential time plots are shown in Fig. 4 and it can be seen that the AP1 and AP2 treated coating exhibited nobler potential than AC and AP3. From 1 h to 24 h (1d) of exposure, the potential shifted towards anodic side for treated coating and it may be attributed due to the formation of AHP on coating surface (Fig. 3 and Fig. S1) while AC Al coating exhibited many more connected pores/defects which make the steel surface as cathode and gives mixed potentia^18^,^57^.

As AHP come in contact with solution started to dissolute the coating and make the electrolytic solution as acidic. Therefore, the coating leads to deteriorate for initial period of exposure. The shifting of AP3 potential towards active side indicates deterioration of AHP film (Fig. 2) in artificial ocean water during initial period of exposure. It may be due the more acidic nature of AP3 treated coating than other treatments i.e. AP1 and AP2. As the exposure period is increased (after 24 h), the corrosion potential of AP1 treated coating ennobled towards cathodic side up to 1008 h (42d) of exposure which owing to the formation of passive film^58^,^59^,^60^. Thereafter it is increased (anodic) up to 1440 h (60d) due to initiation of corrosion. The more active (anodic) potential of AP3 than AC Al coating due to formation of AHP which is active in nature. The potential of AP3 treated coating drastically shifted from −0.646 V to −0.898 V for 1 h to 24 h (1d) and this result indicates the deterioration effect in artificial ocean water. The AP2 and AC Al coating exhibited stabilized potential up to 288 h (12d) of exposure periods thereafter it is shifted towards active side. The AP1 and AP2 coating attributed positive potential than AC and AP3 and it might be due to formation of protective film which stifled the penetration of aggressive ions of artificial ocean water. From potential plots (Fig. 4), it can be seen that the ennobling of potential for AP2, AP3 and AC after 720 h (30d) may be attributed due to deposition of corrosion products on coating surface in artificial ocean water^61^,^62^,^63^,^64^.

#### EIS studies of Al coating in artificial ocean water with exposure periods

**Figure 5:**
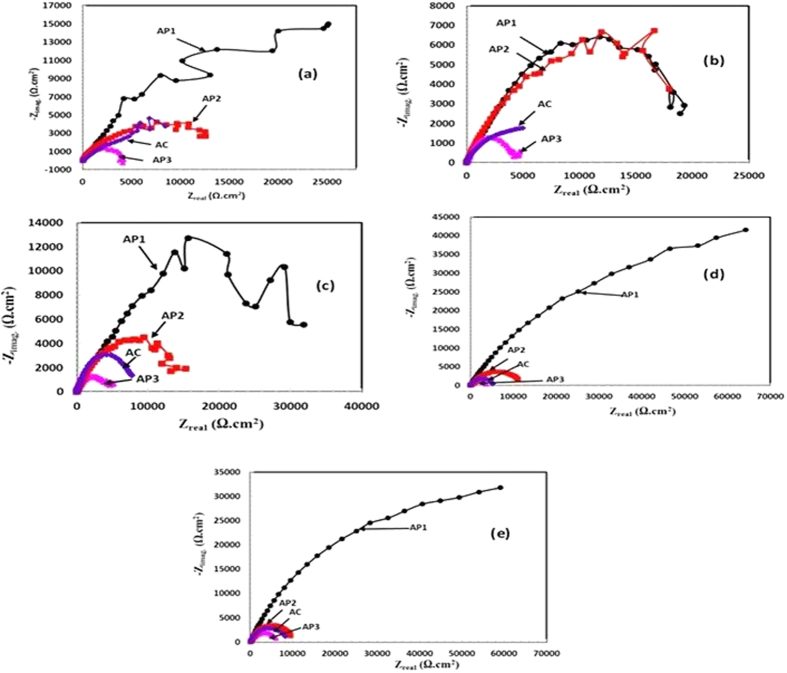
Nyquist plots of treated and AC Al coating applied by arc thermal spray process in artificial ocean water after **(a)** 1 h, **(b)** 1d, **(c)** 8d, **(d)** 42d and **(e)** 60d of exposure.

The evaluation of corrosion resistance performance of treated and AC Al coatings was carried out by EIS studies in artificial ocean water with exposure periods at their OCP. The Nyquist plots of coatings in artificial ocean water are shown in Fig. 5a–e. The semi-circle loops are not well defined in Fig. 5a for AP1 and AP2 coating owing to formation of capacitive loops after 1 h of exposure. It is assumed that the bigger dimensions of semi-circle loop in Nyquist plots of these treated coatings (AP1 and AP2) than AP3 and AC after 1 h of exposure show the corrosion resistance assets in solution^65^. The semi-circle loop of Nyquist plots is characterized by three different process at high, medium and low frequencies which represent solution resistance, charge transfer resistance and double layer capacitance, respectively^62^,^66^,^67^,^68^. The semi-circle loop is also attributed the formation of capacitance during corrosion process^9^. However, the AP1 coating exhibited bigger loop than other coatings for 1 h of exposure (Fig. 5a) in solution which indicate less corrosion^69^,^70^.

The AP1, AP2 and AC coatings are showing identical features in Nyquist plots after 1 h of exposure and the semi-circle loop dimensions are higher than AP3 which suggest high degree of protection. In Nyquist plots for initial periods of exposure (from 1 h to 8d), these coatings exhibited two times constant (Fig. 5a–c), first time constant appeared at high and second at low studied frequencies owing to solution/coating interface for cracked coating and solution/oxide interface for cracked free coating, respectively^38^. The dimensions of semi-circle loops of AC and AP1 coating decreased considerably which seem to deterioration of coating in artificial ocean water up to 24 h (1d) of exposure while AP2 and AP3 remained as such 1 h of exposure (Fig. 5b). The decrease in dimension for AP1 coating is due to formation of very thin layer of AHP. AP1 contains dense, regular and needle like crystalline microstructure (Fig. 1a). This phenomenon attributed that thin layer of AHP react with solution and started to dissolute easily resulted a decrease in polarization resistance of AP1 coating up to 24 h (1d) of exposure.

As exposure period is increased, the trend for formation of semi-circle loop is changed and one very interesting observation is found that the increase in dimension of semi-circle loop for AP1 coating after 8d of exposure in solution (Fig. 5c). This result suggests that after 8d of exposure, the polarization resistance of AP1 is increased significantly which diminishes the corrosion process. The AP3 coating has one small semi-circle loop at low studied frequency which may be attributed due to formation of double layer capacitance up to 8d of exposure. The formation of double layer capacitance represents susceptibility of coating to corrosion which further induce corrosion to continuous exposure in solution.

Longer duration i.e. 42d and 60d of exposure in artificial ocean water reveals the actual performance of treated and AC Al coating for their resistance to corrosion. It can be said that the performance of any coatings or materials would depend on their longer duration of exposure in aggressive environment. Furthermore, the treated and AC Al coating were exposed in artificial ocean water for 42d and 60d and their Nyquist plots are shown Fig. 5d,e. The highest increase in dimension of semi-circle loop is observed in AP1 coating after 42d of exposure and this result suggests that the optimum concentration among all AP solutions for Al coating is 0.1 M. However, a certain exposure period is required where coating can form very protective and adherent transformed compound after proper reaction with artificial ocean water. Other than AP1 coating such as AP2, AP3 and AC coating exhibited decrease in semi-circle loop that is attributing the decrease in polarization resistance. Due to enlargement in semi-circle loop of AP1 coating, others are suppressed and show smaller loops (Fig. 5d). The decrease in dimension of semi-circle loop of AP1 after 60d of exposure is observed (Fig. 5e) and it is attributed due to deterioration of formed compound/corrosion product on coating surface with continuous exposure in solution. This result indicates that AP1 coating start the deterioration after 60d of exposure but attain passivity up to such period.

At high frequency, there is one very small semi-circle loop appeared for AP1 coating which may be due to presence of coating capacitance while at low frequency due to formation of double layer capacitance up to 8d of exposure. After 42d and 60d of exposure, AP1 coating exhibited one bigger loop which may be formation of high resistance capacitive features. On the other hand, AP2 also exhibited same type of characteristics in Nyquist plot as AP1 up to 8d of exposure but smaller in size. Bigger loop suggests high corrosion resistance of coatings while lower in size indicate more susceptible to corrosion^69^,^70^.

The AC coating exhibited same characteristics in shape of Nyquist plot for 1 h of exposure as AP1 and AP2 but smaller in size. The AP3 coating shows two semi-circle loops from 1 h to 8d of exposure at higher and lower studied frequency correspond to coating capacitance and deterioration effect, respectively and it may be due to more concentration of AP solution which induces the coating for deterioration. On the other hand, the AC coating exhibited straight line at low studied frequency in Nyquist plot after 1d (24 h) of exposure owing to deterioration of coating and later it forms corrosion product on surface. An interesting observation is found after 8d of exposure of AC coating, the shapes of Nyquist plots are same as AP3 coating. It means that AP3 and AC coating after 8d to up to 60d of exposure show same characteristics and it is an indication of more deterioration of coating in artificial ocean water.

The AP2 coating exhibits same features throughout the exposure periods (1 h to 60d) in artificial ocean water and the only differences are in dimensions and size of semi-circle loops. This results suggest that AP2 coating would form only one electrical equivalent circuit (EEC) for all exposure periods.

**Figure 6:**
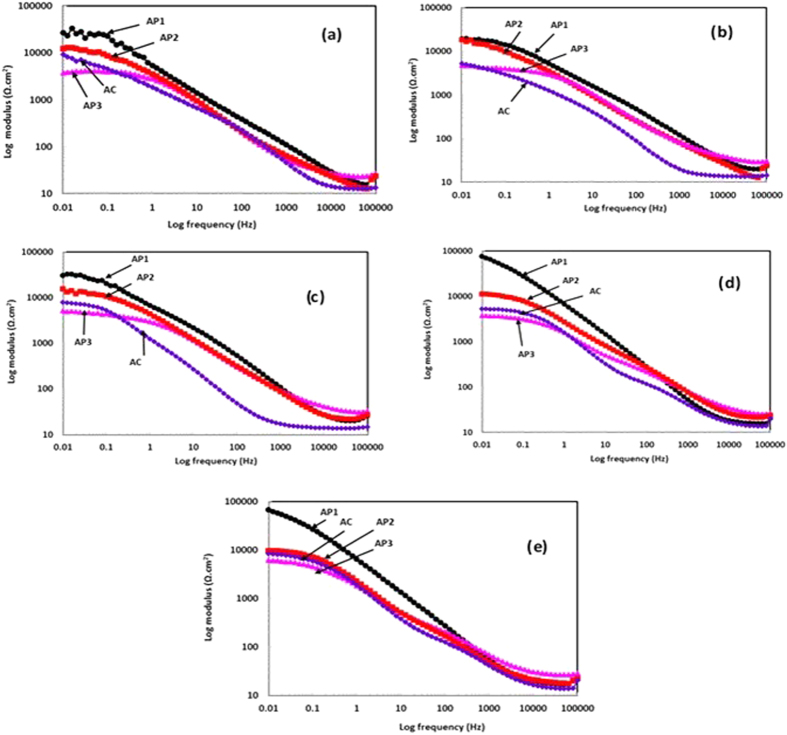
Log modulus-frequency phase Bode plots of treated and AC Al coating applied by arc thermal spray process in artificial ocean water after **(a)** 1 h, **(b)** 1d, **(c)** 8d, **(d)** 42d and **(e)** 60d of exposure.

The coating characteristics are also determined by log modulus-frequency phase Bode plots of treated and AC Al coating and shown in Fig. 6a–e. After 1 h of exposure (Fig. 6a) of coating in artificial ocean water exhibited high degree of impedance value which suggest that these coatings would resist the penetration of aggressive ions of solution towards substrate. During initial (1 h) of exposure, the coating and solution was not reacted properly and require some time to initiate the reaction. However, 1 h is not significant to start the corrosion process of coating and whatever the properties exhibited by coating is shown by such exposure period. The AP1 coating exhibited highest log modulus value followed by AP2, AC and AP3 which mean it would provide more protection to coating than others. The impedance at lowest studied frequency (0.01 Hz) indicates total polarization resistance (*R*_*pore*_) while at the highest frequency (100 kHz) shows solution resistance (*R*_*s*_) and it can be seen from log modulus phase frequency plots that AP1 coating exhibited highest *R*_*pore*_ values.

Owing to continuous exposure of coating in artificial ocean water, the impedance values are decreased due to presence of aggressive ions of solution and water that penetrate from pores/defects and cracks of coating and cause deterioration. However, the decrease in impedance of all coatings are observed (except AP2) after 24 h (1d) of exposure in artificial ocean water (Fig. 6b). The AP1 and AP2 Al coting are exhibited almost same impedance values after 24 h (1d) of exposure but a considerable decrease in impedance of AP3 and AC Al coating is observed (Fig. 6b). Even though the log modulus value for AP3 is lower than AC coating after 24 h (1d) of exposure in solution. It may be the AP3 coating deteriorating more than other coatings in artificial ocean water.

As the exposure periods increases, the impedance value for AP1 coating gradually increases while other coatings decrease after 8d of exposure in artificial ocean water (Fig. 6c). The increase in impedance of AP1 coating may be either transformation of AHP to other phase or formation of adherent, compact and protective corrosion products. The decrease in log modulus impedance of AP2 coating after 8d of exposure attributed the deterioration of coating (Fig. 6c). The decrease in impedance of AP3 coating than AC may be due to more concentration of AHP which is susceptible to corrosion. It may be the sufficient amount of AHP which had formed on AP3 coating and leads to dissolve it and acidified the solution. This result suggests that certain amount of AHP is required for providing proper protection to coating and this observation may be correlated with phase identification by XRD plots (Fig. 3) and Raman spectroscopy (Fig. S1) studies.

The Log modulus frequency Bode plots for coatings after 42d and 60d of exposure in artificial ocean water are shown in Fig. 6d,e. The impedance value for AP1 coating is observed to be the highest after 42d of exposure in artificial ocean water (Fig. 6d). This result is attributed due to formation of very protective, adherent and transformed compound that is able to resist the penetration of aggressive ions of solution and water toward substrate. On the other hand, the other coatings i.e. AP2, AP3 and AC Al coating impedance values are continuously decreased than prior studied exposure periods. The AP2 and AP3 coatings which contain high content of AHP than AP1 and aggressive ions i.e. Cl^−^, CO_3_^−^ and SO_4_^−^ in solution are responsible for deterioration of these coatings.

The Log modulus plot for AP1 coating decreases after 60d of exposure (Fig. 6e) than 42d of exposure and it may be ascribed due to continuous exposure of coating in aggressive i.e. artificial ocean water. Such type of observations is also found for other coatings i.e. AP2, AP3 ad AC. The exact mechanism for reaction of coating in artificial ocean water with exposure periods will be discussed in subsequent paragraphs where we studied the morphology and nature of corrosion products or passive film on coating surface by different analytical techniques.

**Figure 7:**
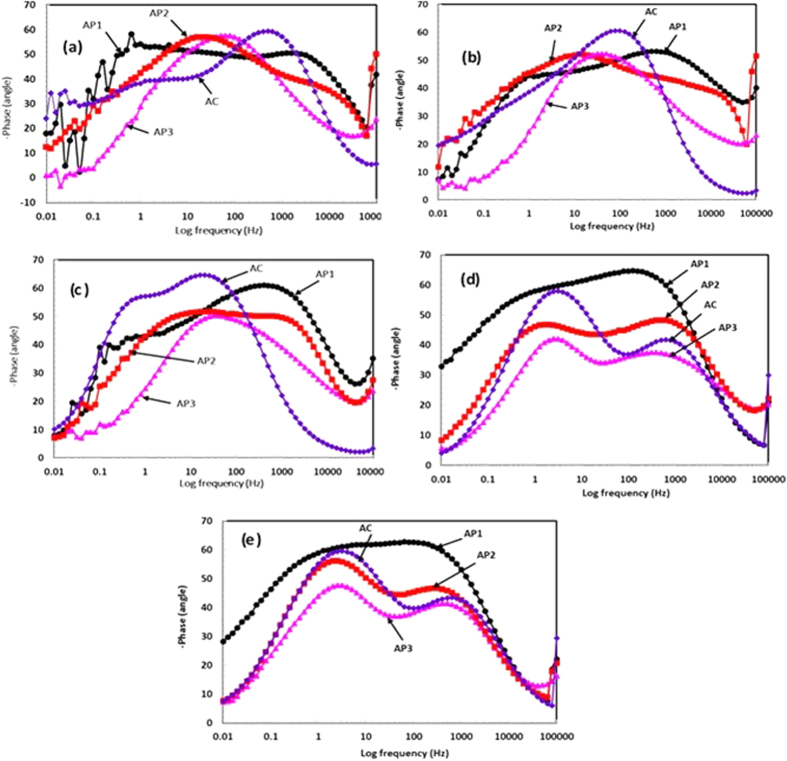
Phase-frequency Bode plots of treated and AC Al coating applied by arc thermal spray process in artificial ocean water after **(a)** 1 h, **(b)** 1d, **(c)** 8d, **(d)** 42d and **(e)** 60d of exposure.

The frequency - phase Bode plots of coatings are shown in Fig. 7a–e with exposure periods in artificial ocean water. The AP1 coating exhibited one capacitive loop (Fig. 7a) between 0.3 Hz to 6 kHz. The formation of this capacitive loop attributed due to formation of compact, uniform and crystalline morphology on coating surface. The other coating revealed two capacitive properties at different studied frequency ranges i.e. middle and low frequency attributed due to presence of double layer or charge transfer resistance and coating capacitance, respectively. For AC coating, the maxima shifted towards high frequency indicate corrosion of coating due to presence of more defects on coating surface^71^. The shifting of maxima at low angle in lower frequency for AP3 than AC Al coating is attributed due to susceptibility of it towards corrosion in artificial ocean water. The shifting of capacitive loop at lower frequency for AP2 coating ascribed by the corrosion resistance (Fig. 7a). The shifting of phase angle towards zero at high studied frequencies suggested that the coating having more defects or cracking^72^.

After 24 h (1d) of exposure of AP1 coating in artificial ocean water exhibited two maxima at different studied frequency i.e. one very distinct at low and another at middle frequency. The maxima at low frequency may be formation of capacitive passive film while at middle frequency attributed due to appearance of charge transfer resistance. The AP2 coating shifted the maxima (−50°) at lower frequency which indicates resistance to corrosion^73^,^74^,^75^. The phase-frequency Bode plots of AP3 and AC coating illustrating the shifting of maxima in middle frequency attributed the double layer capacitance due to deposition of corrosion products. The maxima exhibited at −50° from 100 Hz to 6 Hz for AP3 while −60° from 250 Hz to 25 Hz for AC coating (Fig. 7b).

The characteristics of capacitive loops in phase-frequency Bode plots are distinguished by shifting to them at different studied frequencies range. However, the AP1 coating exhibited one capacitive loop at middle frequency and another one is middle to low frequencies range (Fig. 7c) owing to presence of double layer capacitance and deposition of protective passive film respectively, after 8d of exposure in artificial ocean water^76^,^77^. The corrosion products might be uniformly deposited through all over the coating surface after initiation of corrosion process and resist the penetration of solution towards substrate. The AP2 coating is exhibited one capacitive loop from 2000 Hz to 1 Hz which is not separated by other loop and it seems high resistance to corrosion (Fig. 7c). AP3 coating shows same assets after 8d of exposure as found for 24 h (1d) of exposure in artificial ocean water. AC Al coating shifted maxima at different angles with two capacitive loops in middle frequency region. One capacitive loop at −65° from 60 Hz to 6 Hz and it indicates presence of double layer capacitance while another is due to deposition of corrosion products on coating surface at −55° from 1 Hz to 0.5 Hz (Fig. 7c).

As progress of exposure periods, the AP1 coating exhibited very high degree of protection and it can be seen from shifting of maxima of phase angle at −65° to −60° from 500 Hz to 0.1 Hz, respectively (Fig. 7d) after 42d of exposure. The higher range of covered frequency region suggest that it form very protective uniform passive film on coating surface^76^,^77^. This observation indicates non-ideal capacitor behavior of AP1 coating. This result can be corroborated with Nyquist (Fig. 5d) along with log modulus plots (Fig. 6d) which shows very high degree of protection provided by AP1 coating. AP2 coating after 42d of exposure is also exhibited same trends in phase-frequency plots (Fig. 7d) as AP1 but maxima shifted towards lower angle (approximately −48°). It is well known phenomena that the shifting of maxima towards lower angle (approximately −40°) from high to low studied frequency facilitates corrosion of materials and such type of observation is found in case of AP3 coating after 42d of exposure in artificial ocean water (Fig. 7d). The AC coating is also exhibited two capacitances from 2500 Hz to 300 Hz and 15 Hz to 1 Hz at approximately −40° and −60°, respectively. This phenomena of corrosion process for AP3 and AC coating after 42d of exposure in artificial ocean water is owing to interaction of solution with pores/defects or pits of coating surface^78^. The former shifting attributed due to solution resistance while the later one due to formation of double layer capacitance between coatings pores/defects and solution interface.

After 60d of exposure of all coatings in artificial ocean water indicate deterioration characteristics. This observation indicates that for longer duration of exposure in artificial ocean water started to dissolute the coating. The matter of fact here that coating which would sustain for longer duration is the best among all coatings. The AP1 coating also exhibited high degree of protection than other coatings for their longer duration of exposure. The phase-frequency plots after 60d of exposure for AP1 coating cover maximum range of studied frequency at approximately −63° (Fig. 7e) which attributed that uniform and homogenous film are formed.

**Figure 8:**
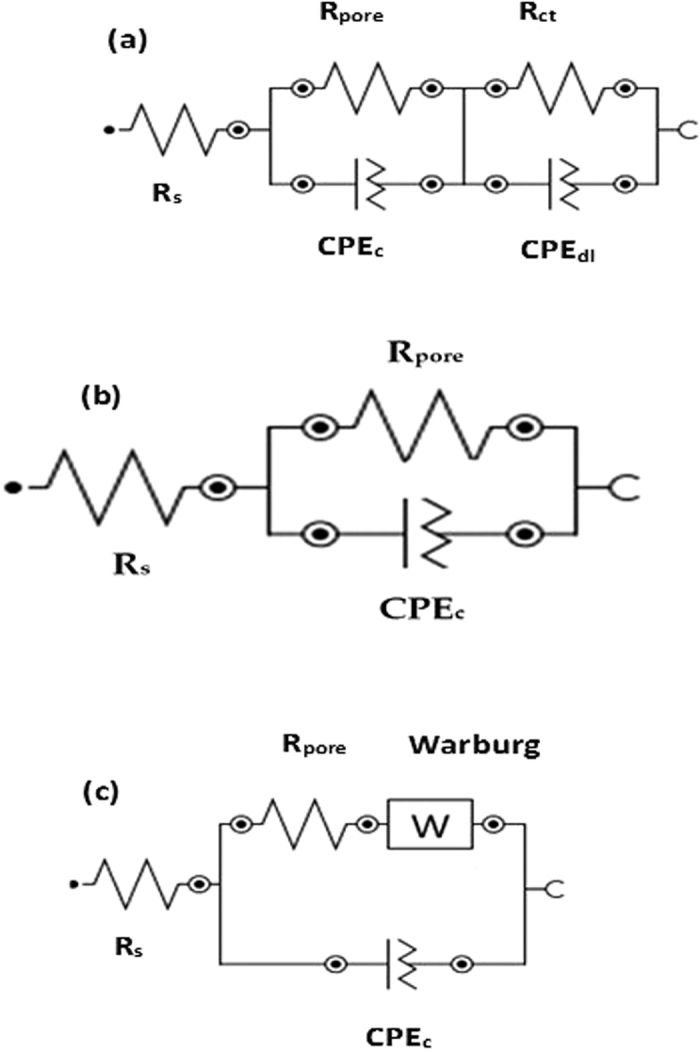
EEC for **(a)** AP1 (1 h to 8d), AP2 (1 h to 60d), AP3 (42d to 60d), AC (1 h, 42d to 60d), **(b)** AP3 (1 h to 8d), AC (8d) and **(c)** AP1 (42d to 60d), AC (1d) Al coating exposed in artificial ocean water.

On the basis of EIS results, equivalent electrical circuit (EEC) has been deduced. It is found that different circuit is proposed due to difference in shape of EIS plots of coating exposed in artificial ocean water with exposure periods. The different EEC are explained for different coating samples with exposure periods. In Fig. 8a, *R*_*s*_*, R*_*pore*_*, CPE*_*c*_*, R*_*ct*_ and CPE_dl_ indicate solution resistance between coating and solution interface, polarization resistance, constant phase element of coating, charge transfer resistance and constant phase element for double layer, respectively. The presence of CPE_dl_ in EEC is due to inhomogeneous surface of coating^79^. The EEC modelled for AP1, AP2 and AC coating after 1 h of exposure in artificial ocean water is shown in Fig. 8a. The coating itself has capacitive properties, therefore presence of *CPE*_*c*_ is found for all studies exposure periods. After exposure of coating in solution, reaction on coating surface would be started hence, again one resistance being appeared which called charge transfer resistance (*R*_*ct*_). Due to presence of *R*_*ct*_ on coating surface, another capacitance appeared which called double layer capacitance of constant phase element (*CPE*_*dl*_). Such type of EEC can be modelled for different duration of exposure of coating in artificial ocean water. This EEC is used for AP1 coating after 1 h to 8d while for AP2 it exhibited all period of exposure. This circuit is also used for AP3 coating after 42d and 60d while for AC coating 1 h, 42d and 60d of exposure.

Figure 8b shows EEC deduced the simple circuit and it contains only *R*_*s*_*, R*_*pore*_ and *CPE*_*c*_. It is common circuit for general electrochemical reaction of materials^8^,^9^,^76^,^80^,^81^,^82^. This circuit is modelled for AP3 coating after 1 h to 8d of exposure because this coating treated with 1 M AP solution and this concentration of AP solution cause localized or pitting corrosion of coating. After 8d of exposure, AC coating started more corrosion and it is due to presence of more pores/defects on coating surface which cause corrosion of coating.

The presence of W (Warburg) impedance in Fig. 8c for AP1 (42d and 60d) and AC (1d) Al coating attributed due to formation of diffusion layer. Since the solution reaction with AP1 coating would transform AHP to a very adherent and protective corrosion layer while AC coating started deterioration due to inherent properties of arc thermal spray coating (Fig. 1d). The solution penetrating from pores/defects of AC coating and started corrosion. The corrosion products may diffuse into the pores/defects and resultant would increase in *R*_*pore*_.

**Figure 9:**
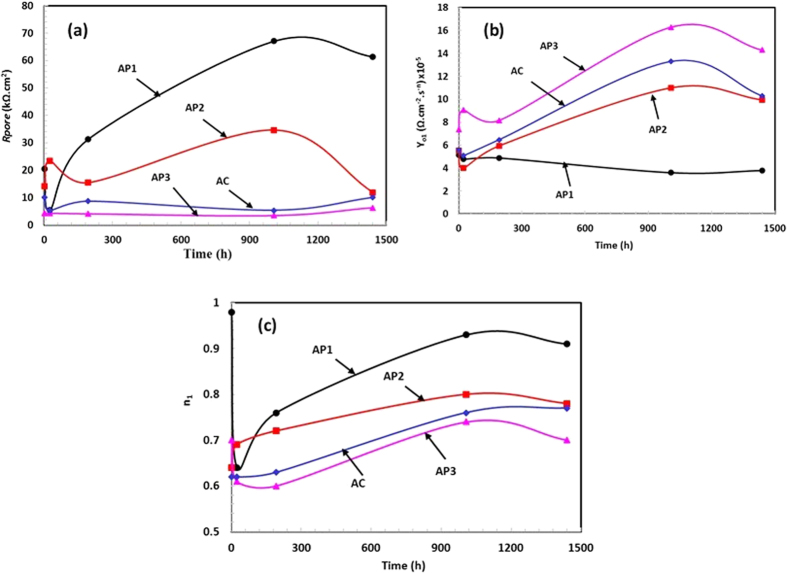
Electrochemical parameters of coating after fitting of EIS data in suitable EEC in artificial water ocean water with different exposure periods **(a)** *R*_*pore*_**(b)** *Y*_*o1*_ and **(c)** n_1_.

After appropriate fitting of EIS plots in suitable EEC, electrochemical parameters were calculated and presented in Table S1 and Fig. 9. It can be seen from Fig. 9a that *R*_*pore*_ of AP1 coating is exhibited maximum for 1 h of exposure followed by AP2, AC and AP3 in artificial ocean water. The *R*_*pore*_ decreases up to 24 h (1d) of exposure except AP2 treated Al coating but AP1 coating increased significantly as exposure period increased and reached maximum after 42d of exposure. The ennobling in *R*_*pore*_ for AP1 coating in artificial ocean water with exposure periods may be formation of protective oxides/phases which stifle the penetration of aggressive ions. In spite of treatment of coating, AP3 exhibited maximum decrease in *R*_*pore*_due to more content of AHP on coating surface which attributed negative impact on performance of treated Al coating.

It is well established that as *R*_*pore*_ increases, the admittance (*Y*_*o*_) decreases and such observations are found in this study too (Fig. 9b). The *Y*_*o*_ increases as time of exposure periods increases due to decrease in *R*_*pore*_ of AP2, AP3 and AC Al coating in artificial ocean water (Fig. 9b). The maximum *Y*_*o*_ is found for AP3 followed by AC, AP2 and AP1 coating and this result corroborating the *R*_*pore*_ results. The increased n (dispersion coefficient) at 0.98 (Fig. 9c) indicates the homogeneity of AP1 surface after 42d of exposure in artificial ocean water. The presence of *CPE*_*dl*_ (Table S1) due to formation of double layer on coating surface which appeared during exposure of coating in artificial ocean water and form thin and homogeneous layer^83^.

As mentioned in Fig. 8a that AP2 and AC coating show same EEC as such AP1 after 1 h of exposure but the *R*_*pore*_ is lesser than AP1 (Fig. 9a). It is general observation that if *Y*_*o*_ is high then *R*_*pore*_ and n should be low. Such type of observation is also found in this study. The *R*_*ct*_ for AP1 coating is less than AP2 coating for 1 h of exposure in artificial ocean water (Table S1) and it may be due to formation of more AHP on AP2 coating surface after treatment with AP solution which causes charge transfer resistance to ingress of aggressive ions of solution. The less *R*_*ct*_ for AC Al coating than AP1 and AP2 after 1 h of exposure is due to presence of more pores/defects on surface.

After 24 h (1d) of exposure, *R*_*pore*_ of AP1, AP3 and AC coating is decreased (Fig. 9a) while AP2 increased but the *R*_*ct*_ of AP1 is increased than AP2 (Table S1). The presence of W in AC coating (Table S1) due to deterioration of it which form diffused layer on coating surface. The high *R*_*ct*_ for AP1 coating may be attributed due to formation of uniform and adherent compound on coating surface after reaction with artificial ocean water. As the exposure periods of coating in artificial ocean water is increased, the *R*_*pore*_ as well as *R*_*ct*_ increased which suggest high degree of protection exhibited by AP1 coating. This result indicates that AP1 coating inhibited the surface for deterioration after 8d of exposure due to transformation of optimum content of AHP i.e. AP1 into other protective compound which fill the pores/defect of coating. This optimum content of AHP react with artificial ocean water and transformed it into other protective compound while other coatings which contain high amount of AHP i.e. AP2 and AP3 exhibited deteriorating feature.

After 42d and 60d of exposure, it is observed that AP1 coating exhibited W impedance which may be due to formation of protective passive film and it properly stifled the corrosion phenomena of coating in artificial ocean water. The W impedance appears due to diffusion of protective film of AP1 coating while for other coatings i.e. AP2, AP3 and AC coating show *R*_*ct*_. After 42d of exposure of AP1 coating in artificial ocean water exhibited highest *R*_*pore*_ during all exposure periods and this result suggests that 42d is the incubation periods which transformed the AHP into other protective compound and passive film while other coatings is decreased continuously with exposure periods. The mechanism of formation of transformed protective compound of coating will be discussed in subsequent paragraph. The *R*_*pore*_ is decreased for AP1 after 60d of exposure which indicates that it started to deteriorate but not as much than other coatings.

From the above results, it can be said that the optimum content of AP solution to provide efficient and effective corrosion resistance in artificial ocean water is 0.1 M. The formation of protective, non-porous and uniform passive film may be explained as


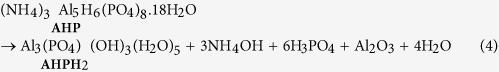


The protective mechanism of Aluminum hydroxide phosphate hydrate [AHPH: Al_3_(PO_4_)_2_(OH)_3_(H_2_O)_5_] can be derived by different analytical techniques which will be discussed in subsequent paragraph under heading of evaluation of corrosion resistance properties of Al coating after exposure in artificial ocean water.

#### Potentiodynamic studies of Al coating in artificial ocean water after 60d of exposure

**Figure 10:**
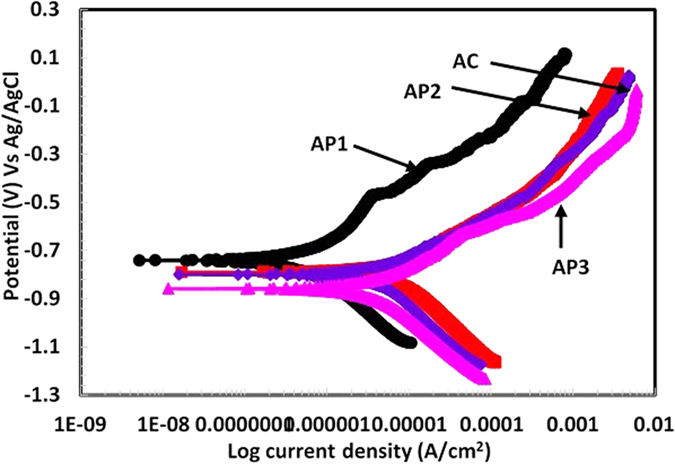
Potentiodynamic plots of treated and AC Al coating applied by arc thermal spray process in artificial ocean water after 60d of exposure.

After 60d of exposure of coatings in artificial ocean water, potentiodynamic studies were carried out to determine their corrosion resistance properties. The potentiodynamic plots of coatings are shown in Fig. 10. The corrosion current and potential of AP1 coating is shifted towards lower and nobler side, respectively which attributed the less corrosion rate and formation of passive film while other coatings is far from AP1 coating^79^. The potential of AP1 coating shifted towards positive side than other coatings suggesting that this coating has better corrosion protection. AP1 coating exhibited passive property during anodic scanning and breakdown potential at −0.468 V Vs Ag/AgCl. The presence of breakdown potential in AP1 coating attributed due to formation of another phase which block the pores/defects and deposited on the coating surface whereas the other coatings exhibiting active potential. AP2 and AC coatings are showing almost identical characteristics during anodic scanning while AP3 is different. The anodic current density of AP1 coating increases slightly while others are rapidly increased towards higher side. Although, the increase in current density during anodic scanning reveals pitting tendency of AP3 coating. AP3 coating exhibits a limiting current during anodic scanning around 4.28 mA/cm^2^ from −0.230 V to −0.051 V Vs Ag/AgCl while other do not show such behavior. Such type of observation for AP3 coating is due to more deterioration of coating where cation migrate from coating surface to solution and reduces the diffusion of oxygen. This observation indicates that the mass transfer at coating surface is stifled by oxide film due to formation of more thick corrosion products. Thus, it is not necessary that the thick corrosion product is uniform and does not have any defects. This observation shows that AP3 coating having defective corrosion products, therefore, the current density increased sharply. However, in this case, the corrosion product may have cracks or defects where the penetration of aggressive ions of solution is allowed and induces corrosion process. On the other hand, the cathodic polarization of coatings indicate that the AP3 coating exhibit lesser current density while AP2 and AC show higher than this coating. This phenomenon of AP3 coating attributed due to hydrogen evaluation reaction while AC and AP2 coating due to oxygen reduction reaction^38^. Due to this process of AP2 and AC coating cathodic current increased while AP3 coating treated with more content of AP solution where H^+^ ion liberated during reaction (equation 1). The hydrogen evaluation reaction dominates on AP3 coating over oxygen reduction reaction. Therefore, the cathodic current is found to be less while in case of AC and AP2 it is vise-versa. In case of AP1 coating very protective passive/oxide film is to be formed which reduces the cathodic reaction resultant less cathodic current is observed. The more reduction reaction occurred on AP2 and AC coating attributed that whatever oxide films formed on these coating surfaces was reduced and deposited on coating surface which block the porosity of coating but in AP3 coating H_2_ gas entrapped in pores/defect which later enhances the corrosion during anodic scanning.

The corrosion current density (*I*_*corr*_) and *E*_*corr*_ of coatings are calculated after fitting of potentiodynamic plots in Tafel region and presented in Table 2. The *I*_*corr*_ of AP1, AP2, AP3 and AC Al coatings are 0.96, 7.83, 8.65 and 8.48 μA/cm^2^ and *E*_*corr*_ are −0.735, −0.781, −0.850 and −0.793 V Vs Ag/AgCl, respectively in artificial ocean water after 60d of exposure. The *I*_*corr*_ and *E*_*corr*_ of AP3 is to be highest and active, respectively compare to other coatings which indicate high corrosion rate. The active *E*_*corr*_ of AP3 indicates anodic dissolution of coating and it may be due to more content of AHP which susceptible to dissolve the coating in artificial ocean water. The artificial ocean water contains Cl^−^, CO_3_^−^ and SO_4_^−^ ions and these ions are very aggressive which is responsible for deterioration of AP3 coating from initial to longer duration of exposure. The *R*_*pore*_ is also very important parameter to evaluate the coating performance in aggressive environment. The more *R*_*pore*_ indicate less corrosion rate of coating and AP1 is exhibiting 87.16 kΩ.cm^2^ followed by 8.38 kΩ.cm^2^, 8.23 kΩ.cm^2^ and 8.16 kΩ.cm^2^ for AP2, AC and AP3, respectively (Table 2). The AP1 exhibiting almost 10 times greater *R*_*pore*_ than other coatings in artificial ocean water after 60d of exposure. This result indicates that the 0.1 M AP solution play a vital role to control the corrosion of Al coating applied by arc thermal spray process in artificial ocean water even after 60d of exposure. 1.0 M AP solution induces the corrosion of coating and this result suggests that more than 0.5 M AP solution is not good for corrosion protection of Al coating. Therefore, it is very important to determine the protective properties of corrosion products which were formed on coating surface after potentiodynamic studies in artificial ocean water by other techniques. However, we will describe these techniques in subsequent paragraphs.

After potentiodynamic studies of coatings, solution analysis was performed to determine the leached content of Al from coating surface. The leached Al from coating surface of AP1, AP2, AP3 and AC are 0.037 ppm (0.047 ppm/cm^2^), 0.039 ppm (0.050 ppm/cm^2^), 0.220 ppm (0.282 ppm/cm^2^) and 0.115 ppm (0.148 ppm/cm^2^), respectively. The leaching of Al from AP1 coating is lowest while AP3 is highest after potentiodynamic studies in artificial ocean water after 60d of exposure. This result is also corroborating with EIS and potentiodynamic studies which suggest that more content i.e. 1.0 M AP solution is having detrimental effect for Al coating to fill the pores/defect of coating.

#### Evaluation of corrosion resistance properties of coating after exposure in artificial ocean water

**Figure 11:**
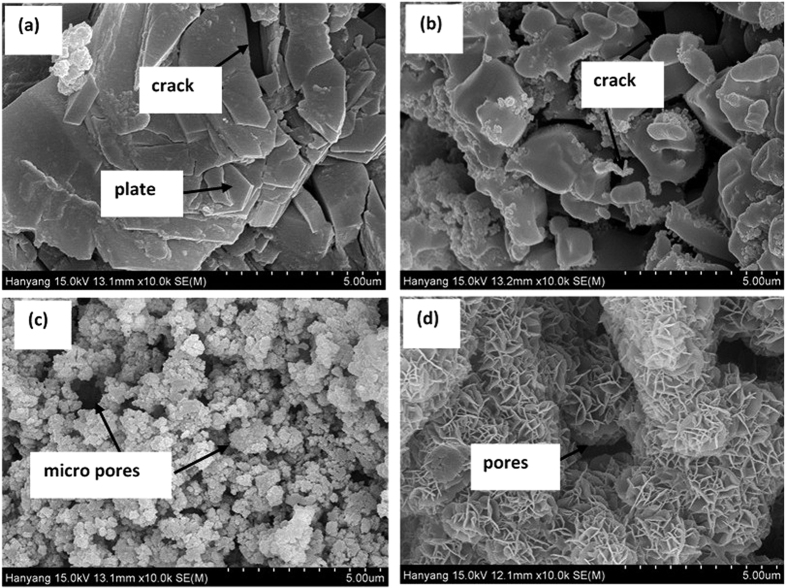
SEM images of Al coating **(a)** AP1, **(b)** AP2, **(c)** AP3 and **(d)** AC after potentiodynamic studies in artificial ocean water for 60d of exposure.

The morphology of corrosion products of treated and AC Al coating after potentiodynamic studies in artificial ocean water for 60 d of exposure were performed by FE-SEM. The FE-SEM are shown in Fig. 11 and their EDS analysis in Table 3. The potentiodynamic experiment is destructive electrochemical technique in which surface is being disturbed. Therefore, it is observed that all coating surface are exhibiting cracks and pores formation. However, the AP1 coating surface exhibited compact plate like microstructure with minimum cracking. The corrosion products morphology of AP1 is regular and layer to layer which inhibited the coating surface against corrosion (Fig. 11a). As the concentration of AP solution treatment is increased, the morphology of corrosion products which were formed on coating surface are drastically changed after potentiodynamic studies in artificial ocean water. AP2 coating is also exhibited as such AP1 but with more number of cracking and defects (Fig. 11b) which is responsible for higher corrosion rate than AP1. AP3 coating surface is showing uniform, micro pores formation with different size, finely structured and coagulated (Fig. 11c) types corrosion products which facilitate the ingress of aggressive ions of artificial ocean water resultant highest corrosion rate among all studied coatings. AC Al coating exhibited macro size pores (Fig. 11d) formation with fine petals and net like corrosion products which stifle the ingress of artificial ocean water than AP3. A significant appearance of white deposits on AP3 and AC Al coating surface may be due to deposition of salt or charging effect of corrosion products (iron oxide might come out on coating from substrate through fine pores of corrosion products of coating after potentiodynamic experiments)^84^. The surface was not chemically cleaned, only washed with distilled water for one time and during such process corrosion product residue and salt remain attached to surface^85^. Such type of observation is not found in AP1 and AP2 treated coating samples which revealed that there is no chance for deposition or occurrence of salt or iron oxides.

The EDS analysis of corrosion products revealed the content of elements which were present after potentiodynamic studies in artificial ocean water for 60d of exposure on coating surface and these are presented in Table 3. The content of C is sufficient amount in corrosion products which may come by carbonation of solution (ASTM D1141). The other elements i.e. Na, Mg, S, Cl and Ca are also found in EDS analysis as very less amount on AP1, AP2 and AC Al coating while AP3 contain 15.53 wt.% Na and 19.96 wt.% Cl in corrosion product. The presence of high content of Cl on AP3 surface than other coatings attributed due to high content of AHP (Fig. 3 and Fig. S1) which has greater affinity to react with Cl than low content of AHP (AP1 and AP2). This result indicates that higher content of AHP react with Cl^−^ ion of solution and induces the corrosion of AP3 coating. The presence of above mentioned elements in corrosion products is due to their respective salts in artificial ocean water (ASTM D1141). From Table 3 it can be seen that the content of N and P are gradually decreases as content of treatment of AP solution increased in corrosion products. The N and P content are decreased in corrosion products of coating which may be due to reaction of treated coating with artificial ocean water. These elements might be dissolved significantly in artificial ocean water after potentiodynamic studies (equation 4). One very interesting observation is noted here that the content of Al in Table 3 is decreased as content of treatment of AP is increased. This result is strongly recommending that the leaching of Al is more as content of AP is increased on coating surface after potentiodynamic studies. This result is corroborating the result of ICP-MS analysis of solution which were performed after potentiodynamic studies and it is found that the leached Al content for AP3 is the highest and AP1 is the least.

**Figure 12:**
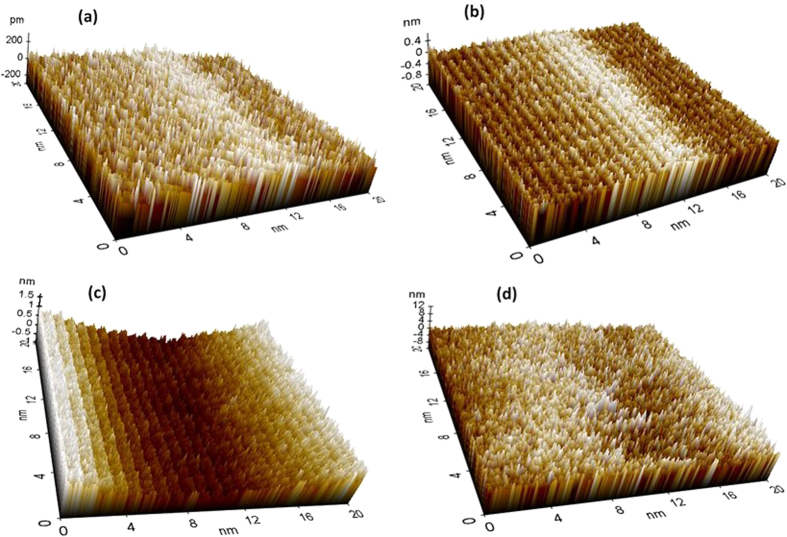
AFM (topography images: 3D) of Al coating **(a)** AP1, **(b)** AP2, **(c)** AP3 and **(d)** AC after potentiodynamic studies in artificial ocean water for 60d of exposure.

The AFM of corrosion products are shown in Fig. 12. The small, uniform and regular needle like corrosion products are deposited on AP1 coating (Fig. 12a) surface while AP2 coating (Fig. 12b) exhibits bigger and smaller with different sizes which may show more roughness than AP1. The uniform deposition of corrosion products stifles the ingress of solution towards coating surface which reduces the corrosion phenomena. The roughness of corrosion products predicts and control the deterioration process for later age. AP3 coating exhibit valley, up and down corrosion product deposition. It can be seen from Fig. 12c that AP3 coating shows more valley region on selected area of image which attributed due to deposition of more roughen corrosion products. The AC surface (Fig. 12d) showing different types of deposition of corrosion products as coagulated, uneven and different size of needle like orientation which enhances the corrosion process. The roughness of corrosion products was measured by using software fitting and these are 0.05, 0.10, 0.25 and 0.21 nm for AP1, AP2, AP3 and AC coating, respectively. The lowest calculated roughness of AP1 coating reveals that corrosion products are protecting effectively to the surface for further deterioration^86^,^87^. The roughness and appearance of corrosion products indicate that the AP1 coating attributed high resistance to corrosion while AP3 more susceptible to corrosion for longer duration of exposure in artificial ocean water and cannot control the corrosion. This result indicates that more content of AP solution is not favorable to control the corrosion and fill the porosity of Al coating. This result is also express that the AP1 coating contain more adherent, protective and less active corrosion products which may be sparingly soluble. Therefore, other techniques are required to corroborate the AFM results. The findings of AFM results are corroborating with FE-SEM and it is confirmed that the corrosion products which were formed on AP1 coating surface exhibited high degree of protection against corrosion in artificial ocean water after potentiodynamic studies for 60d of exposure.

**Figure 13:**
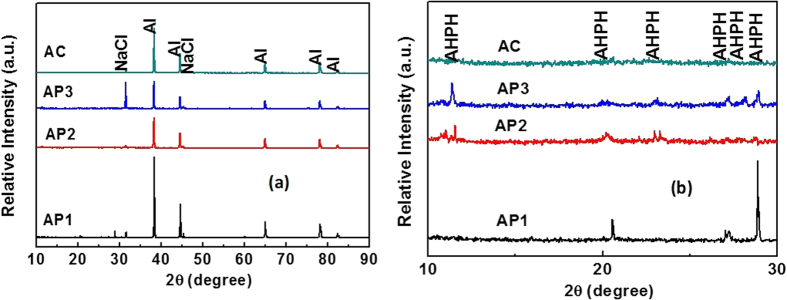
XRD plots **(a)** full range 2θ = 10° to 90° **(b)** 2θ = 10^o^ to 30^o^ of treated and AC Al coating applied by arc thermal spray process in artificial ocean water after 60d of exposure.

After potentiodynamic studies of coatings, it is very important to determine their corrosion resistance properties by different technique. Therefore, the XRD of corrosion products were performed and shown in Fig. 13. The Al peak is dominant in XRD plots of Fig. 13a and some other peaks are also present but their intensity is very less. The presence of more intense peaks of Al in corrosion products for AP1 coating (Fig. 13a) revealed that the content of Al is more than other coatings. The leaching of Al from AP1 coating is less than other coating after potentiodynamic studies for 60d of exposure in artificial ocean water which is also confirmed by EDS of corrosion products and ICP analysis of solution. For full range of XRD scanning lower intensity peaks are not identified properly and suppressed, therefore, 2θ = 10° to 30° is plotted in Fig. 13b. The AHPH peaks are clearly seen in Fig. 13b and the intensity of this phase in AP1 is most intense than other corrosion products. The presence of NaCl on corrosion products may be due to exposure of coatings in artificial ocean water which contain NaCl. This NaCl might be deposited on coating surface and detected by XRD. The NaCl peak intensity in AP3 is higher than other corrosion products and it may be the affinity of it with AHP is more (more content of AHP in AP3). The Al_2_O_3_ might be present in corrosion products with very trace amount (equation 4) but it concentration is beyond the limit of XRD detection. Hence, it was not detected by XRD.

The calculated V_f_ of corrosion products which were formed on coating surface after 60d of exposure in artificial ocean water are shown in Table S2. From Table S2 it can be observed that the AP1 coating surface exhibiting more concentration of AHPH than others treated coating. The V_f_ of AP1 is 5.57% followed by AP2 and AP3 with 5.07% and 4.46%, respectively. One very interesting observation can be seen from Table S2 that on AP3 coating surface NaCl is very high and this result seems that this coating surface influences the deposition of Cl^−^ ion which stimulated the corrosion process of it. The V_f_ of NaCl for AP1, AP2, AP3 and AC Al coatings are 7.07, 10.71, 43.26 and 14.86%, respectively. It may be that more AHP (Fig. 3 and Fig. S1) influences the affinity of NaCl and lead to more deposition on coating surface resultant initiation of localized or pitting corrosion. Hence, the AHPH concentration is less on AP3 than AP2 and AP1 coating surface. The V_f_ of Al are 87.36, 84.22, 52.28 and 85.14% for AP1, AP2, AP3 and AC Al coating, respectively.

Figure S2. represent the Raman spectra of treated and AC Al coating applied by arc thermal spray process in artificial ocean water after 60d of exposure. The peaks around 450 cm^−1^ belongs bending vibration while 1120 cm^−1^ to asymmetric stretching of P-O-P group on treated coating corrosion products. These peaks are absent in AC Al coating which confirm that in this coating there is no phosphate containing group. The P-O-P bond ascribed the presence of AHPH phase. The treated coating exposed in artificial ocean water for 60d are exhibiting Raman shift around 980 cm^−1^ which attributed due to asymmetric bending vibration of Al-O-Al group^53^,^54^,^55^. The Raman peak at 720 cm^−1^ attributed for symmetric stretching ν_as_Al-O-P group. These peaks are absent in AC Al coating. The presence of η-Al_2_O_3_ in AC as well as treated Al coating at 1260 cm^−1^ in Raman spectra (Figs S1 and S2) ascribing that it present on Al surface even after treatment and exposure in artificial ocean water after 60d but it is not detected by XRD due to limitation of this instrument. Al and NaCl are not detected by Raman spectroscopy because it is not sensitive to Raman spectroscopy. The presence of AHPH in corrosion products confirm the formation of protective, non-porous and adherent layer. The AHPH may be thermodynamically stable and sparingly soluble in solution.

## Discussion

**Figure 14:**
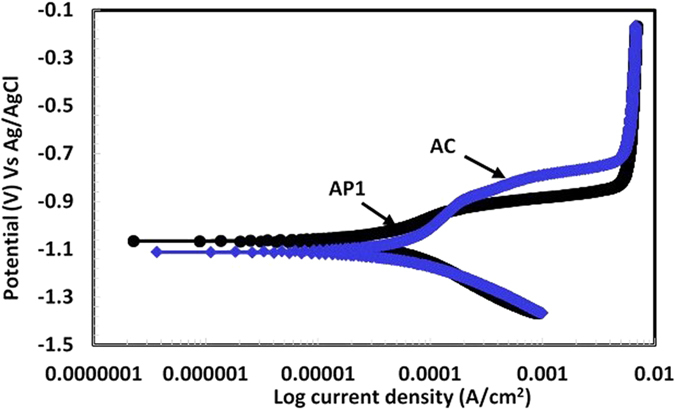
Coating exposing them in 1 wt.% NaCl for 7d of dip/dry thereafter potentiodynamic studies were carried out in artificial ocean water after 1 h of exposure.

Since from the above results it is found that AP1 coating performing superior than other coatings. Therefore, it was decided to perform the accelerated experiment of coating by exposing AP1 and AC Al coating in 1 wt.% NaCl solution for 7d to dip and dry alternately. This experiment had performed to generate the sufficient corrosion product and to know their characteristics in artificial ocean water. The coating was immersed in 1 wt.% NaCl solution for 12 h and kept 12 h outside from solution at 25 °C (±1 °C). This experiment was continued for 7d to generate the proper corrosion product on coating surface thereafter potentiodynamic studies were performed in artificial ocean water after 1 h of exposure. The potentiodymnamic plots for AP1 and AC Al coating are shown in Fig. 14 and electrochemical parameters were extracted after fitting of plots in Tafel region and shown in Table S3. Both coating exhibited passivation tendency due to formation of thick corrosion products which passivate the coating.

The AP1 coating exhibited nobler potential than AC after accelerated experiment to expose the coatings in 1 wt.% NaCl solution for dip/dry test which attributed that the nature of formation of corrosion products is very adherent and protective. This film reduces the penetration of aggressive ions of artificial ocean water towards the coating surface. However, Fig. 14 shows the limiting current density around 4.69 mA/cm^2^ for both the samples and it may due to deposition of thick corrosion products which reduces the mass transfer reaction resultant constant current during anodic scanning of coatings up to −0.198 V Vs Ag/AgCl. The cathodic region of both samples exhibited same phenomena and indicate oxygen reduction reaction but at more cathodic side the AC coating shows more cathodic current. This result indicates that AC pronounced by oxygen reduction reaction of oxides (corrosion products) resultant formation of less protective oxide film. The *E*_*corr*_ and *I*_*corr*_ for AP1 coating are −1.040 V and 103.65 μA/cm^2^ while AC Al coating −1.130 V and 165.21 μA/cm^2^, respectively (Table S3). The *E*_*corr*_ is noble and *I*_*corr*_ is less for AP1 coating while AC Al coating exhibiting active *E*_*corr*_ and higher *I*_*corr*_ which indicate that the nature of corrosion product on treated coating surface is protective and adherent with minimum porosity. The corrosion product formed on AC Al coating after dip/dry test was in more amount than AP1 coating which can be seen by naked eye (Fig. not mentioned). This observation indicates that whatever corrosion product was formed on AP1 coating surface are very protective and block the pores/defects of coating significantly.

**Figure 15:**
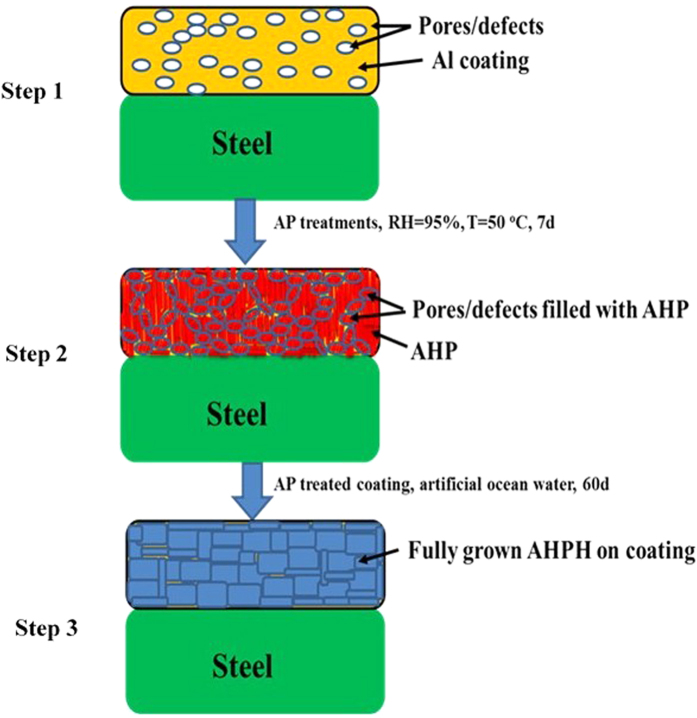
Illustration of schematic diagram for corrosion process of AP treated Al coating exposing them in artificial ocean water after 60d.

The pore sealing properties of AP treated Al coating is derived by schematic and shown in Fig. 15. The pores/defects formation on coating surface of arc thermal spray coating process is inherent properties. It can be seen from step 1 of Fig. 15 that coating surface showing different sizes of interconnected pores/defects which is inherent property of arc thermal spray process. This coating is not corrosion resistance due to formation of pores/defects on coating surface, hence, it was decided to fill/seal the pores/defects of coating by treating them using economical and eco-friendly chemical.

The AP is the best chemical which significantly reduces the porosity and roughness of AC Al coating (Figs 1 and 2). Only chemical treatment is not enough to fill the porosity so that the coating was exposed in humidity chamber at 95% relative humidity (RH) and 50 °C for 7d to simulate the environmental condition.

As the AP solution concentrations was increased, the morphology of treated Al coating is gradually changed and can be seen from FE-SEM images. In more concentration of AP solution, the coating bearing cracks on surface which may induce the corrosion of treated Al coating (Fig. 1c). From step 2 of Fig. 15, it can be seen that the pores/defects are uniformly filled by AHP which was formed after treatment and in specific condition.

The morphology of such coating can be seen from Fig. 1 where different content of AP solution was treated on Al coating. Thereafter, it was decided to evaluate the performance of treated as well as AC Al coating in artificial ocean water and this solution is very aggressive due to its composition which contain Cl^−^, SO_4_^−^ and CO_3_^−^ ions. Since, the treated coating withstands in such solution then it can be supposed that the treated coating can perform in any aggressive environments.

After treatment of Al coating, it was exposed in artificial ocean water and different electrochemical studies were performed with exposure periods. From initial period of exposure, treated coating was performed better than AC Al coating except AP3 and it found that AP1 is exhibited superior among all treated coating for longer duration of exposure. The formed AHP on coating surface is unstable and non-protective but the morphology at 0.1 M AP solution treated coating (AP1) exhibited uniform, crystalline structure while others are having some cracks/defects. Therefore, it formed different morphology of coating and when it was exposed in artificial ocean water. The more content of AHP (Fig. 3 and Fig. S1) on coating surface started to dissolute and reduce the pH of artificial ocean water solution due to liberation of H^+^ resultant it causes induced corrosion for initial period of exposure. From step 3 of Fig. 15 it can be contributed that after 60d of exposure of AP1 treated coating exhibited uniform, plate and regular morphology (Fig. 11) with less roughness (Fig. 12) which attributed due to formation of fully grown and sparingly soluble AHPH on coating surface (Fig. 13 and Fig. S2). The compact and plate like morphology which resist the penetration of aggressive ions towards coating surface resultant enhanced the corrosion resistance of AP1 coating for longer duration of exposure.

From the above results, the following conclusions can be summarized:

1. The treatment of AP solution on Al coating applied by arc thermal spray process exhibited improved corrosion resistance in artificial ocean water with exposure periods.
2. High content of AP solution showed deteriorating effect to Al coating. Therefore, the optimum concentration of AP solution for the best corrosion resistance of Al coating on steel surface in artificial ocean water is 0.1 M. This concentration of AP solution exhibited almost 10 times greater corrosion resistance than other coatings.
3. By increasing the content of AP, the solution become acidic which cause deterioration of Al coating. Therefore, 1.0 M AP containing solution decreases its performance than AC Al coating in artificial ocean water with exposure periods.
4. FE-SEM result reveals the formation of uniform, needle like and crystalline morphology of treated Al coating with 0.1 M AP solution which attributed the superior performance of Al coating.
5. Kinetics and mechanism of corrosion process of Al coating by EIS and potentiodynamic studies suggest that AP1 has performed in greater extent than other coatings in artificial ocean water.
6. After potentiodynamic studies of coatings, solution analyses of leached Al content revealed the least amount of Al for AP1 coating which exhibited the magnified corrosion resistance.
7. The morphology of coatings and corrosion products were studied with FE-SEM and AFM. These techniques show that high content of AP solution exhibited higher corrosion owing to acidic nature of solution which prone to deterioration of coating. High content of AP solution develops cracks and more roughness on coating surface.
8. The more content AHP on AP3 coating reacted with artificial ocean water and dissolved. Therefore, the solution become acidic resultant decrease in polarization resistance of coating.
9. The XRD and Raman spectroscopy of coatings and corrosion products indicate the formation of AHP in coating while AHPH in corrosion products. AHPH is sparingly soluble and thermodynamically stable due to formation of phosphate bond with Al.

## Additional Information

**How to cite this article**: Lee, H.-S. *et al*. An effective and novel pore sealing agent to enhance the corrosion resistance performance of Al coating in artificial ocean water. *Sci. Rep.* **7**, 41935; doi: 10.1038/srep41935 (2017).

**Publisher's note:** Springer Nature remains neutral with regard to jurisdictional claims in published maps and institutional affiliations.

## Supplementary Material

Supplementary Information

## Figures and Tables

**Table 1:** EDS analysis of treated and AC Al coating.

Coating ID	Element (wt.%)				
	N	O	Al	P	Total
AP1	5.12	22.04	62.47	10.37	100.00
AP2	6.09	39.47	33.06	21.38	100.00
AP3	8.35	50.74	12.32	28.60	100.00
AC	—	2.32	97.68	—	100.00

**Table 2:** Electrochemical parameters of Al coating were extracted after fitting of potentiodynamic plots in Tafel regions after 60 days of exposure in artificial ocean water.

Coating ID	Electrochemical parameters		
	*E*_*corr*_ (V) Vs Ag/AgCl	*I*_*corr*_(μA/cm^2^)	*R*_*pore*_(kΩ.cm^2^)
AP1	−0.735	0.96	87.16
AP2	−0.781	7.83	8.38
AP3	−0.850	8.65	8.16
AC	−0.793	8.48	8.23

**Table 3:** EDS analysis of Al coating after potentiodynamic studies in artificial ocean water for 60d of exposure.

Coating	Element (wt.%)										
ID	C	N	O	Na	Mg	Al	P	S	Cl	Ca	Total
AP1	9.54	4.71	17.59	4.01	4.55	44.01	8.76	1.14	4.44	1.25	100
AP2	13.39	4.62	30.54	4.38	3.13	27.77	7.06	2.11	4.36	2.64	100
AP3	8.45	3.95	15.50	15.38	3.30	20.34	6.75	4.35	19.96	2.02	100
AC	13.32	—	44.15	4.51	1.16	26.73	—	3.92	4.48	1.73	100

## References

[b1] Jandin G., Liao H., Feng Z. Q. & Coddet C. Correlations between operating conditions, microstructure and mechanical properties of twin wire arc sprayed steel coatings. Mater. Sci. Eng. A 349, 298–305 (2003).

[b2] Guilemany J. M., Miguel J. M., Armada S., Vizcaino S. & Climent F. Use of scanning white light interferometry in the characterization of wear mechanisms in thermal-sprayed coatings. Mater. Charact. 47, 307–314 (2001).

[b3] Pawlowski L. The Science and Engineering of Thermal Spray Coatings, 2nd ed., John Wiley & Sons Ltd, West Sussex (2008).

[b4] Chaliampalias D., Vourlias G., Pavlidou E., Stergioudis G., Skolianos S. & Chrissafis K. High temperature oxidation and corrosion in marine environments of thermal spray deposited coatings. Appl. Surf. Scie. 255, 3104–3111 (2008).

[b5] Choe H.-B., Lee H.-S. & Shin J.-H. Experimental study on the electrochemical anti corrosion properties of steel structures applying the arc thermal metal spraying method. Materials 7, 7722–7736 (2014). 10.3390/ma7127722 PMC5456417 28788271

[b6] Krepski R. P. Thermal spray: coating applications in the chemical process industries. Published for the Materials Technology Institute of the Chemical Process Industries (1993).

[b7] Paredes R. S. C., Amico S. C. & M.d’Oliveira A. S. C. The effect of roughness and pre-heating of the substrate on the morphology of aluminum coatings deposited by thermal spraying. Surf. Coat. Technol. 200, 3049–3055 (2006).

[b8] Lee H.-S., Singh J. K., Ismail M. A. & Bhattacharya C. Corrosion resistance properties of aluminum coating applied by arc thermal metal spray in SAE J2334 solution with exposure periods. Metals 6, 1–15 (2016).

[b9] Lee H. S., Singh J. K. & Park J. H. Pore blocking characteristics of corrosion products formed on Aluminum coating produced by arc thermal metal spray process in 3.5wt.% NaCl solution. Constr. Build. Mater. 113, 905–916 (2016).

[b10] Lee H. S., Park J.-H., Singh J. K. & Ismail M. A. Protection of reinforced concrete structure of waste water treatment reservoirs with stainless steel coating using arc thermal spraying technique in acidified water. Materials 9, 1–20 (2016). 10.3390/ma9090753 PMC5457078 28773875

[b11] ASTMA 775 M-93. “Specification for Epoxy Coated Reinforcing Steel Bars” (West Conshohocken. PA: ASTM International. (1993).

[b12] Smith L. L., Kessler R. J.& Powers R. G. “Corrosion of Epoxy-Coated Rebars In a Marine Environment: Transportation Research Circular no. 403. Transportation Research Board. National Research Council. Washington. DC. March (1993).

[b13] Li Y., Liu J., Duan J. & Hou B. Thermally sprayed aluminium and zinc coatings for tidal zone cathodic protection of offshore platform pile leg. Mater. Perfor. 45, 16–20 (2006).

[b14] Zhang X. . Mitigation of corrosion on Magnesium alloy by pre-designed surface corrosion. Sci. Rep. 59, 17399 (2015). 10.1038/srep17399 PMC4663789 26615896

[b15] Xue C.-H., Bai S. & Jia S.-T. Robust, self-healing, super hydrophobic fabrics prepared by one-step coating of PDMS and Octadecylamine. Sci. Rep. 6, 27262 (2016). 27264995 10.1038/srep27262 PMC4893697

[b16] Wang G. . Robust superhydrophobic surface on Al substrate with durability, corrosion resistance and ice-phobicity. Sci. Rep. 6, 20933 (2016) 26853810 10.1038/srep20933 PMC4745080

[b17] Gill B. J. Method of reducing porosity in thermal spray coated and sintered articles, US patent No. US20070098975A1. published on 3^rd^ May (2007).

[b18] Xu X., Lu Y., Liu H., Wu Y. & Liu Q. Aqueous pore sealing agent improving PCB coating oxidation-resistance and corrosion-resistance properties and method for using same, US patent No. US2014314967. published on 23^rd^ October (2014).

[b19] Jiang Q. . Corrosion behavior of arc sprayed Al–Zn–Si–RE coatings on mild steel in 3.5 wt% NaCl solution. Electrochim. Acta 115, 644–656 (2014).

[b20] Valdez B., Kiyota S., Stoytcheva M., Zlatev R. & Bastidas J. M. Cerium-based conversion coatings to improve the corrosion resistance of aluminium alloy 6061-T6. Corros. Scie. 87, 141–149 (2014).

[b21] Jeong C., Lee J., Sheppard K. & Choi C.-H. Air-impregnated nanoporous anodic aluminum oxide layers for enhancing the corrosion resistance of Aluminum. Langmuir 31, 11040−11050 (2015). 26393523 10.1021/acs.langmuir.5b02392

[b22] Chidambaram D., Clayton C. R. & Halada G. P. Interactions of the components of Chromate conversion coating with the constituents of Aluminum alloy AA2024-T3. J. Electrochem. Soc. 151, B151−B159 (2004).

[b23] Zhao J., Franke l. G. & McCreery R. L. Corrosion protection of untreated AA-2024-T3 in chloride solution by a chromate conversion coating monitored with Raman Spectroscopy. J. Electrochem. Soc. 145, 2258−2264 (1998).

[b24] Xia L. & McCreery R. L. Chemistry of a chromate conversion coating on Aluminum alloy AA2024-T3 probed by vibrational spectroscopy, J. Electrochem. Soc. 145, 3083−3089 (1998).

[b25] Kuznetsova A., Burleigh T. D., Zhukov V., Blachere J. & Yates J. T. Electrochemical evaluation of a new type of corrosion passivation layer: Artificially produced Al_2_O_3_ films on Aluminum. Langmuir 14, 2502−2507 (1998).

[b26] Barik R. C., Wharton J. A., Wood R. J. K., Stokes K. R. & Jones R. L. Corrosion, erosion and erosion−corrosion performance of plasma electrolytic oxidation (PEO) deposited Al_2_O_3_ coatings. Surf. Coat. Technol. 199, 158−167 (2005).

[b27] Hintze P. E. & Calle L. M. Electrochemical properties and corrosion protection of organosilane self-assembled monolayers on Aluminum 2024-T3. Electrochim. Acta 51, 1761−1766 (2006).

[b28] Hughes A. E., Cole I. S., Muster, Tim H. & Varley R. J. Designing green, self-healing coatings for metal protection. NPG Asia Mater. 2, 143–151 (2010).

[b29] Sankara Narayanan T. S. N. Surface pretreatment by phosphate conversion coatings – A review. Rev. Adv. Mater. Sci. 9, 130–177 (2005).

[b30] Shibli S. M. A. & Chacko F. Development of nano TiO_2_-incorporated phosphate coatings on hot dip zinc surface for good paintability and corrosion resistance. Appl. Surf. Sci. 257, 3111–3117 (2011).

[b31] Jiang Cong-cong . Alumina induced crystal Refinement and enhanced corrosion resistance of phosphate chemical conversion coating on 35CrMnSi Steel. ECS Electrochem. Letters 4, C23–C25 (2015).

[b32] ASTM D1141, Standard practice for the preparation of substitute ocean water, 100 Barr Harbor Drive, PO Box C700, West Conshohocken, PA 19428-2959, USA (2003).

[b33] ASTM D4541, Standard Test Method for Pull-Off Strength of Coatings Using Portable Adhesion Testers; ASTM International: West Conshohocken, PA, USA (2009).

[b34] Jiang Q. . Electrochemical corrosion behavior of arc sprayed Al–Zn–Si–RE coatings on mild steel in 3.5 wt% NaCl solution. Trans. Nonferrous Met. Soc. China 24, 2713–2722 (2014).

[b35] Kotoka R. . Electrochemical corrosion behavior of silver doped tricalcium phosphate coatings on magnesium for biomedical application. Surf. & Coat.Technol. 292, 99–109 (2016).

[b36] Hesaraki S., Safari M. & Shokrgozar M. A. Development of beta-tricalcium Phosphate/sol-gel derived bioactive glass composites: Physical, Mechanical, and *In Vitro* biological evaluations. J. Biomed. Mater. Res. Part B-Appl. Biomater. 91B, 459–469 (2009). 10.1002/jbm.b.31422 19507141

[b37] Abedi E. E., Hamidreza S., Golozar M. A., Mostaghimi J. & Pershin L. Study of Corrosion Behavior of Arc Sprayed Aluminum Coating on Mild Steel. J. Therm. Spray Technol. 21, 1195–1202 (2012).

[b38] Imaz N., Ostra M., Vidal M., Diez J. A., Sarret M. & Garcia-Lecina E. Corrosion behaviour of chromium coatings obtained by direct and reverse pulse plating electrodeposition in NaCl aqueous solution. Corros. Scie. 78, 251–259 (2014).

[b39] Tang J. L. & Zuo Y. Study on corrosion resistance of palladium films on 316L stainless steel by electroplating and electroless plating. Corros. Sci. 50, 2873–2878 (2008).

[b40] Park J., Lee E.-J. & Kwon S.-H. Influence of surface treatment of polyimide film on adhesion enhancement between polyimide and metal films. Bull. Korean Chem. Soc. 28, 188–192 (2007).

[b41] Singhbabu Y. N., Sivakumar B., Singh J. K., Bapari H., Pramanick A. K. & Sahu R. K. Efficient anti-corrosive coating of cold-rolled steel in a seawater environment using an oil-based graphene oxide ink. Nanoscale 7, 8035–8047 (2015). 25869204 10.1039/c5nr01453k

[b42] Yadav M. & Kumar S. Experimental, thermodynamic and quantum chemical studies on adsorption and corrosion inhibition performance of synthesized pyridine derivatives on N80 steel in HCl solution. Surf. Interface Anal. 46, 254–268 (2014).

[b43] Walter R. & Kannan M. B. Influence of surface roughness on the corrosion behaviour of magnesium alloy. Mater. Desig. 32, 2350–2354 (2011).

[b44] Hong T. & Nagumot M. Effect of surface roughness of early stages of pitting corrosion of Type 301 stainless steel. Corros. Scie. 39, 1665–1672 (1997).

[b45] Lagno F. & Demopoulos G. P. The Stability of hydrated Aluminium phosphate, AlPO_4_·1.5H_2_O. Environ. Technol. 27, 1217–1224 (2006). 17203603 10.1080/09593332708618735

[b46] Blanton T. N., Barnes C. L. & Lelental M. The effect of X-ray penetration depth on structural characterization of multiphase Bi-Sr-Ca-Cu-0 thin films by X-ray diffraction techniques. Physica C 173, 152–158 (1991).

[b47] Guleryuz H. & Cimenoglu H. Effect of thermal oxidation on corrosion and corrosion–wear behaviour of a Ti–6Al–4V alloy. Biomaterials 25, 3325–3333 (2004). 14980427 10.1016/j.biomaterials.2003.10.009

[b48] Lin D. J., Lin J. H., Chern & Ju C. P. Structure and properties of Ti–7.5Mo–xFe alloys. Biomaterials 23, 1723–1730 (2002). 11950042 10.1016/s0142-9612(01)00233-2

[b49] Zhang L. C., Shen Z. Q. & Xu J. Glass formation in a (Ti, Zr, Hf)–(Cu, Ni, Ag)–Al high-order alloy system by mechanical alloying. J. Mater. Res. 18, 2141–2149 (2003).

[b50] Lu P.-J., Huang S.-C., Chen Y.-P., Chiueh L.-C. & Shih D. Y.-C. Analysis of titanium dioxide and zinc oxide nanoparticles in cosmetics. J. Food Drug Analys. 23, 587–594 (2015). 10.1016/j.jfda.2015.02.009 PMC9351801 28911719

[b51] Yang H., Wen J., Quan M. & Wang J. Evaluation of the volume fraction of nanocrystalsdevitrified in Al-based amorphous alloys. J. Non-Cryst. Solids 355, 235–238 (2009).

[b52] Ehtemam-Haghighi S., Liu Y., Cao G. & Zhang L.-C. Influence of Nb on the β → α″ martensitic phase transformation andproperties of the newly designed Ti–Fe–Nb alloys. Mater. Scie. Eng. C 60, 503–510 (2016). 10.1016/j.msec.2015.11.072 26706557

[b53] John A., Philip D., Morgan K. R. & Devanarayanan S. IR and Raman spectra of two layered aluminium phosphates Co(en)_3_Al_3_P_4_O_16_·3H_2_O and [NH_4_]_3_[Co(NH_3_)6]_3_[Al_2_(PO_4_)_4_]_2_·2H_2_O. Spectrochim. Acta A 56, 2715–2723 (2000). 10.1016/s1386-1425(00)00314-0 11145338

[b54] Hinchcliffe A. J. & Ogden J. S. Infrared spectra and molecular parameters of matrix-isolated Gallium(I), Indium(I), and Thallium(I) Oxides (Ga_2_O, In_2_O, and T1_2_O). J. Chem. Soc. D ., 1053–1054 (1969).

[b55] Hinchcliffe A. J. & Ogden J. S. Matrix isolation studies on the Gallium-Indium-Oxygen system. Infrared spectra and structures of molecular Ga_2_0, ln_2_0, and InOGa. J. Phys. Chem. 77, 2537–2544 (1973).

[b56] Reyes-Lópeza S. Y., Acuña R. S., López-Juárez R. & Rodríguez J. S. Analysis of the phase transformation of aluminum formate Al(O_2_CH)_3_ to α-alumina by Raman and infrared spectroscopy. J. Ceram. Proces. Res. 14, 627–631 (2013).

[b57] Maeva D. D. E., Leshchinsky E. & Maev R. G. Corrosion protection of light alloys using low pressure cold spray. J. Therm. Spray Technol. 21, 304–313 (2012).

[b58] Moran A. L. & Shaw B. A. *In Situ* Evaluation of Oxide Formation in Aluminum Thermal Spray Coatings. J. Electrochem. Soc. 135, 2773–2774 (1988).

[b59] Hurlen T. & Haijg A. T. Corrosion and passive behavior of Aluminum in weakly alkaline solution. Electrochem. Acta 29, 1833–1838 (1984).

[b60] Liu Y. . Protective Film Formation on AA2024-T3 Aluminum Alloy by Leaching of Lithium Carbonate from an Organic Coating. J. Electrochem. Soc. 163, C45–C53 (2016).

[b61] Campo M. . Corrosion resistance of thermally sprayed Al and Al/SiC coatings on Mg. Surf. Coat. Technol. 203, 3224–3230 (2009).

[b62] Liu C., Bi Q. & Matthews A. E. I. S. Comparison, Performance of PVD TiN and CrN coated mild steel in 0.5 N NaCl aqueous solution. Corros. Sci. 43, 1953–1961 (2001).

[b63] Verdian M. M., Raeissi K. & Salehi M. Electrochemical impedance spectroscopy of HVOF-sprayed NiTi intermetallic coatings deposited on AISI, 1045 steel. J. Alloys Compd. 507, 42–46 (2010).

[b64] Wang Y., Tian W., Zhang T. & Yang Y. Microstructure, spallation and corrosion of plasma sprayed Al_2_O_3_-13%TiO_2_ coatings. Corros. Sci. 51, 2924–2931 (2009).

[b65] Zeng R. C., Qi W. C., Zhang F., Cui H. Z. & Zheng Y. F. *In vitro* corrosion of Mg–1.21Li– 1.12Ca–1Y alloy. Prog. Nat. Sci.: Mater. Int. 24, 492–499 (2014).

[b66] Rincon O. de . Evaluating Zn, Al and Al-Zn coatings on carbon steel in a special atmosphere. Constr. Build. Mater. 23, 1465– 1471 (2009).

[b67] Verdian M. M., Raeissi K. & Salehi M. Corrosion performance of HVOF and APS thermally sprayed NiTi intermetallic coatings in 3.5% NaCl solution. Corros. Sci. 52, 1052–1059 (2010).

[b68] Yang D., Liu C., Liu X., Qi M. & Lin G. EIS diagnosis on the corrosion behavior of TiN coated NiTi surgical alloy. Curr. Appl. Phys. 5, 417–421 (2005).

[b69] Ishizaki T., Masuda Y. & Teshima K. Composite film formed on magnesium alloy AZ31 by chemical conversion from molybdate/phosphate/fluorinate aqueous solution toward corrosion protection. Surf. Coat. Technol. 217, 76–83 (2013).

[b70] Wen J. B., Ma J. J. & He J. G. Al-base sacrificial anode material for corrosion protection ; Chemical Industry Press: Beijing, China, p. 67–79 (2012).

[b71] Freire L., Carmezim M. J., Ferreira M. G. S. & Montemor M. F. The electrochemical behaviour of stainless steel AISI 304 in alkaline solutions with different pH in the presence of chlorides. Electrochim. Acta 56, 5280–5289 (2011).

[b72] Abreu C. M. . High frequency impedance spectroscopy study of passive films formed on AISI 316 stainless steel in alkaline medium. J. Electroanal. Chem. 572, 335–345 (2004).

[b73] Stefanov P., Atanasova G., Stoychev D. & Marinova T. S. Electrochemical deposition of CeO_2_ on ZrO_2_ and Al_2_O_3_ thin films formed on stainless steel. Surf. Coat. Technol. 180–181, 446–449 (2004).

[b74] Dongrui Y., Omar R. & Homero C. FeCO_3_ layer evolution for API 5L X52 steel in carbon dioxide-saturated NaCl brine in the presence of 1-decyl-3-methylimidazolium chloride. Corros. Sci. 87, 40–50 (2014).

[b75] Verdian M. M., Raeissi K. & Salehi M. Electrochemical Impedance Spectroscopy of HVOF-Sprayed NiTi Intermetallic Coatings Deposited on AISI 1045 Steel. J. Alloy. Compd. 507, 42–46 (2010).

[b76] Bajat J. B. . Corrosion protection of aluminium pretreated by vinyltriethoxysilane in sodium chloride solution. Corros. Sci. 52, 1060–1069 (2010).

[b77] Trdan U. & Grum J. SEM/EDS characterization of laser shock peening effect on localized corrosion of Al alloy in a near natural chloride environment. Corros. Scie. 82, 328–338 (2014).

[b78] Heakal F. El-Taib, Tantawy N. S. & Shehta O. S. Influence of chloride ion concentration on the corrosion behavior of Al-bearing TRIP steels. Mater. Chem. Phys. 130, 743–749 (2011).

[b79] Yao Y. W., Zhou Y., Zhao C. M., Han Y. X. & Zhao C. X. Preparation and corrosion resistance property of Molybdate conversion coatings containing SiO_2_ nanoparticles. J. Electrochem. Soc. 160, C185–C188 (2013).

[b80] Gudic S., Radosevic J. & Kliskic M. Study of passivation of Al and Al-Sn alloys in borate buffer solutions using electrochemical impedance spectroscopy. Electrochim. Acta 47, 3009–3016 (2002).

[b81] Martin F. J., Cheek G. T., O’Grady W. E. & Natishan P. M. Impedance studies of the passive film on aluminium. Corros. Sci. 47, 3187–3201 (2005).

[b82] Liu Y. J., Wang Z. Y. & Ke W. Study on influence of native oxide and corrosion products on atmospheric corrosion of pure Al. Corros. Sci. 80, 169–176 (2014).

[b83] Wan Zhixin . Improved corrosion resistance and mechanical properties of CrN hard coatings with an atomic layer deposited Al_2_O_3_ Interlayer. Appl. Mater. Interfaces 7, 26716−26725 (2015). 10.1021/acsami.5b08696 26554497

[b84] Feliu S. (Jr) & Llorente I. Corrosion product layers on magnesium alloys AZ31 and AZ61: Surface chemistry and protective ability. Appl. Surf. Scie. 347, 736–746 (2015).

[b85] Walton C. A., Martin H. J., Horstemeyer M. F. & Wang P. T. Quantification of corrosion mechanisms under immersion and salt- spray environments on an extruded AZ31 magnesium alloys. Corros. Sci. 56, 194–208 (2012).

[b86] Yoo B., Shin Ki R., Hwang D. Y., Lee D. H. & Shin D. H. Effect of surface roughness on leakage current and corrosion resistance of oxide layer on AZ91 Mg alloy prepared by plasma electrolytic oxidation. Appl. Surf. Scie. 256, 6667–6672 (2010).

[b87] Sasaki K. & Burstein G. T. The generation of surface roughness during slurry erosion-corrosion and its effect of the pitting potential. Corros. Scie. 38, 2111–2120 (1996).

