# Supplementary material for: An effective and novel pore sealing agent to enhance the corrosion resistance performance of Al coating in artificial ocean water
Source: Sci Rep. 2017 Feb 3;7:41935. doi: 10.1038/srep41935 (PMC5291225; doi:10.1038/srep41935)
Supplement: Supplementary Information [file srep41935-s1.pdf]

An effective and novel pore sealing agent to enhance the corrosion resistance performance of Al coating in artificial ocean water

Han-Seung Lee[1], Jitendra Kumar Singh[1, 2, *], Mohamed A. Ismail[3]

[1]Department of Architectural Engineering, Hanyang University, 1271 Sa 3-dong, Sangrok-gu, Ansan 426-791, Korea

[2]Department of Chemistry, Indian Institute of Engineering Science and Technology (IIEST), Shibpur, Howrah 711 103, West Bengal, India

[3]Department of Civil and Construction Engineering, Faculty of Engineering and Science, Curtin University Sarawak, CDT 250, 98009 Miri, Sarawak, Malaysia

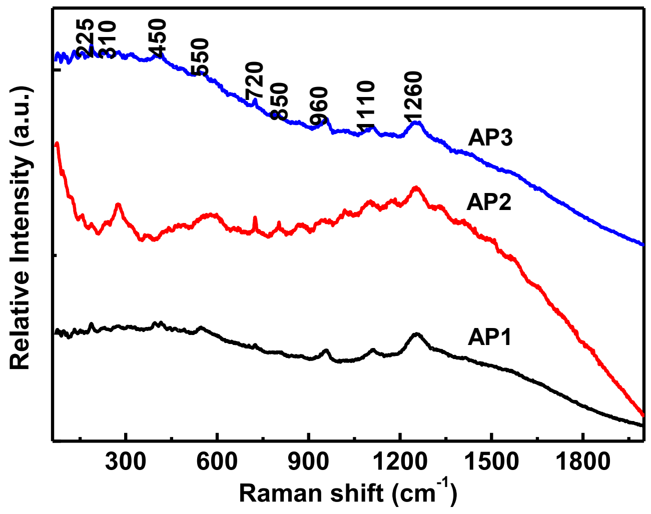

Figure S1. Raman spectra of treated and AR Al coating applied by arc thermal spray process

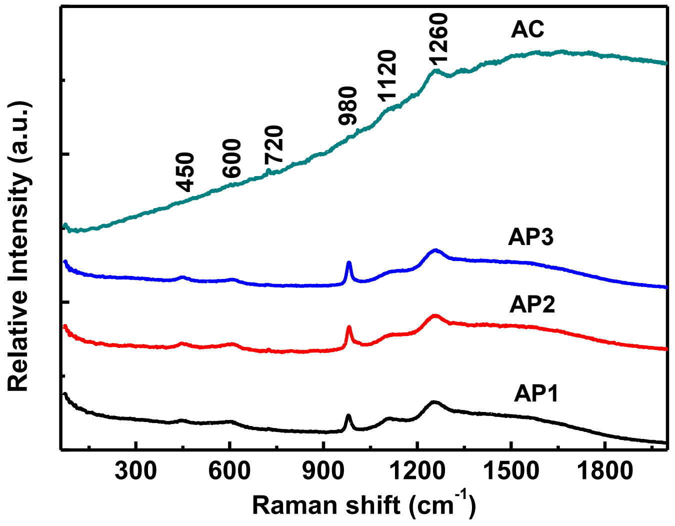

Figure S2. Raman spectra of treated and AC coating applied by arc thermal spray process in artificial ocean water after 60d of exposure

Table S1. EEC parameters of treated and AC coating calculated after fitting of electrochemical electrical circuit

Time	Coating ID	R_s (Ω)	R_{ct} (kΩ)	CPE_{dl} Y_{o2} (1x10^{-5}) ($\Omega^{-1}cm^{-2}.s^{-n}$)	n_2	W (1x10^{-5}) ($\Omega.cm^2.s^{0.5}$)
1h	AP1	8.50	4.88	6.22	0.58	-
	AP2	9.39	12.95	1.45	0.81	-
	AP3	22.14	-	-	-	-
	AC	10.79	0.39	7.43	0.55	-
1d	AP1	9.07	15.50	7.07	0.85	-
	AP2	8.38	12.81	9.46	0.82	-
	AP3	30.63	-	-	-	-
	AC	10.28	-	-	-	279.10
8d	AP1	14.40	2.94	2.32	0.71	-
	AP2	16.63	0.04	2.56	0.61	-
	AP3	25.86	-	-	-	-
	AC	13.38	-	-	-	-
42d	AP1	13.89	-	-	-	8.03
	AP2	15.75	0.26	7.86	0.64	-
	AP3	18.62	0.37	5.34	0.73	-
	AC	13.06	0.09	11.00	0.53	-
60d	AP1	15.13	-	-	-	11.7
	AP2	6.75	0.18	8.03	0.68	-
	AP3	23.7	0.19	5.49	0.69	-
	AC	13.87	0.09	8.89	0.55	-

Table S2. V_f (%) of different phases present on coating surface after potentiodynamic studies in artificial ocean water for 60d of exposure

Coating ID		V_f (%)	
	AHPH	NaCl	Al
AP1	5.57	7.07	87.36
AP2	5.07	10.71	84.22
AP3	4.46	43.26	52.28
AC	0.00	14.86	85.14

Table S3. Electrochemical parameters were extracted after fitting of potentiodynamic plots in Tafel regions after 1h of exposure in artificial ocean water for 7d of dip/dry in 1% NaCl solution.

Coating ID		Electrochemical parameters	
	E_{corr} (V) Vs OCP	I_{corr} (μA/cm^2)	R_{pore} (kΩ.cm^2)
AP1	-1.040	103.65	1.01
AC	-1.130	165.21	0.75
